# Preclinical Evaluation of 2-Aminobenzothiazole Derivatives: In Silico, In Vitro, and Preliminary In Vivo Studies as Diabetic Treatments and Their Complications

**DOI:** 10.3390/molecules30163427

**Published:** 2025-08-20

**Authors:** Natalia Reyes-Vallejo, Miguel Valdes, Adelfo Reyes-Ramírez, Juan Andres Alvarado-Salazar, Alejandro Cruz, Erik Andrade-Jorge, Jessica Elena Mendieta-Wejebe

**Affiliations:** 1 Laboratorio de Biofísica y Biocatálisis, Sección de Estudios de Posgrado e Investigación, Escuela Superior de Medicina, Instituto Politcnico Nacional, Plan de San Luis y Salvador Díaz Mirón s/n, Casco de Santo Tomás, Miguel Hidalgo, Mexico City 11340, Mexico; reyes.natalia4@gmail.com (N.R.-V.); andres.alvarado.salazar@comunidad.unam.mx (J.A.A.-S.); 2 Unidad de Investigación Médica en Farmacología, UMAE Hospital de Especialidades 2 Piso CORSE Centro Médico Nacional Siglo XXI, Instituto Mexicano del Seguro Social, Av. Cuauhtémic 330, Col. Doctores, Mexico City 06720, Mexico; 3 Laboratorio de Inmunología, Departamento de Sistemas Biológicos, Universidad Autónoma Metropolitana Unidad Xochimilco, Calz. del Hueso 1100, Coapa, Villa Quietud, Coyoacán, Mexico City 04960, Mexico; 4 Laboratorio de Síntesis Farmacéutica, Unidad Multidisciplinaria de Investigación Experimental Zaragoza, FES Zaragoza-Universidad Nacional Autonoma de México, Campus II. Batalla 5 de Mayo s/n, Ejército de Oriente Zona Peñón, Iztapalapa, Mexico City 09230, Mexico; adelfo.reyes@zaragoza.unam.mx; 5 Laboratorio de Química Supramolecular y Nanociencias, Unidad Profesional Interdisciplinaria de Biotecnología, Departamento de Ciencias Básicas, Instituto Politécnico Nacional, Av. Acueducto s/n, Colonia Barrio La Laguna Ticomán, Mexico City 07340, Mexico; alcralmx@hotmail.com; 6 Laboratorio de Investigación en Bioquímica, Sección de Estudios de Posgrado e Investigación, Escuela Superior de Medicina, Instituto Politécnico Nacional, Plan de San Luis y Salvador Díaz Mirón s/n, Casco de Santo Tomás, Miguel Hidalgo, Mexico City 11340, Mexico; andrade136@hotmail.com

**Keywords:** benzothiazole, molecular docking, PPAR-γ, aldose reductase, antihyperglycemic

## Abstract

Type 2 diabetes is a multifactorial disease characterized by chronic hyperglycemia, insulin resistance, oxidative stress, inflammation, and dyslipidemia, factors that contribute to the development of long-term complications. In this context, the 2-aminobenzothiazole scaffold has emerged as a promising candidate due to its broad spectrum of biological properties. In this study, we performed a multidisciplinary evaluation of benzothiazole derivatives (**5a**–**d**, **8a**–**d**, **11a**–**d**, and **12c**–**d**), starting with the in silico prediction of their properties, along with molecular docking against aldose reductase (ALR2) and peroxisome proliferator-activated receptor gamma (PPAR-γ). All compounds complied with the main rules of pharmacological similarity and optimal affinity, highlighting **8d** (ΔG = −8.39 kcal/mol for ALR2 and −7.77 kcal/mol for PPAR-γ). Selected compounds from families **C** and **D** were synthesized in moderate yields (~60%) and showed low acute oral toxicity (LD_50_ > 1250 mg/Kg). Compounds **8c** and **8d** inhibited ALR2 at concentrations below 10 µM. In vivo studies using a streptozotocin-induced diabetic rat model with a high-fat diet revealed that compound **8d** produced sustained antihyperglycemic effects and reduced insulin resistance, dyslipidemia, and polydipsia, without inducing hepatotoxicity or displaying intrinsic antioxidant or anti-inflammatory activity. These findings suggest that **8d** is a promising candidate for further development in diabetes-related therapeutic strategies.

## 1. Introduction

The structure of 2-aminobenzothiazole has gained increasing interest in recent research due to its presence in a wide variety of compounds, both natural and synthetic, with multiple biological activities, among which its antidiabetic potential stands out. However, its specific application in the treatment of diabetes has not yet been deeply explored [1].

Diabetes mellitus (DM) is a chronic degenerative non-transmissible disease that represents a priority public health problem. Currently, it affects 588.7 million people worldwide [2] and has a significant impact on quality and life expectancy since sustained glycemic imbalance induces cellular alterations as oxidative stress and chronic inflammation, which over time lead to serious and irreversible damage to different organs, the known diabetic complications [3,4,5]. Despite the availability of multiple treatments on the market, the focus is exclusively on glycemic control, without addressing other associated metabolic disorders, which limits their ability to prevent micro- and macrovascular complications [5,6]. Furthermore, the clinical and therapeutic needs of patients vary widely, and it is sometimes difficult to maintain them within therapeutic goals due to the limitations of existing antidiabetic agents [7,8,9].

Against this backdrop, there is still a need to find new adjuvant therapeutic options that effectively and safely reduce the progression of this disease and its long-term complications through research and the design of molecules based on biologically active structures that can have multiple beneficial effects on the body, such as benzothiazoles [10,11,12,13,14,15]. One of the most relevant new therapeutic targets currently is the ALR2 enzyme. This enzyme is the one that regulates the polyol pathway, whose activation favors sorbitol accumulation, as well as an increase in oxidative stress and inflammation, key phenomena in the progression of microvascular complications such as diabetic neuropathy, retinopathy, and nephropathy. Therefore, its inhibition represents a promising strategy to prevent or mitigate these complications [16,17].

In this context, numerous ALR2 inhibitors have been developed with varying results [17]. The most successful has been epalrestat, the only one to have obtained clinical approval, and it is currently used in countries such as Japan, India, and China [18]. This compound has been shown to reduce neuropathic pain by improving nerve conduction, with a favorable safety profile [19]. In contrast, other more potent inhibitors in vitro studies, such as zopolrestat, failed to advance beyond the initial clinical phases due to the hepatotoxic effects observed in humans and the lack of solid clinical evidence of therapeutic efficacy [20]. This background highlights the importance of designing new ALR2 inhibitors with a better pharmacological profile and lower toxicity.

One of the structural families with the greatest potential in this field is benzothiazoles since specific derivatives, such as guanidine benzothiazoles, have shown antihyperglycemic and hypolipidemic effects in experimental models, apparently through a mechanism similar to pioglitazone (PGZ), which is associated with their agonist effect on PPAR-γ and is considered a key molecular target in the control of type 2 diabetes (T2D) due to its important role in promoting glucose uptake in peripheral tissues, adipocyte re-modeling, and insulin sensitivity, among other metabolic and non-metabolic functions [15,21]. Furthermore, certain 2-aminobenzothiazole derivatives have been identified as capable of inhibiting ALR2 (e.g., zopolrestat), suggesting a potential dual effect: glycemic control and protection against diabetic complications. The similarity in the structural requirements for both activities (PPAR-γ agonism and ALR2 inhibition) makes these compounds promising candidates for the development of multifunctional drugs [16,17].

The selection of the present benzothiazole derivatives incorporating amino acid fragments was based on rational design criteria to optimize their interaction with both targets. Initially, the objective was to introduce carbonyl groups into the new derivatives, given that PPAR-γ ligands, such as thiazolidinediones, typically contain this structural feature, while ALR2 inhibition requires specific interactions with carboxylic groups [17,22].

The inclusion of amino acid fragments attempts to improve the pharmacokinetic properties of the compounds, taking advantage of their structural similarity with endogenous biomolecules and facilitating the variation of substituents without modifying the general synthesis conditions [23].

**Figure 1:**
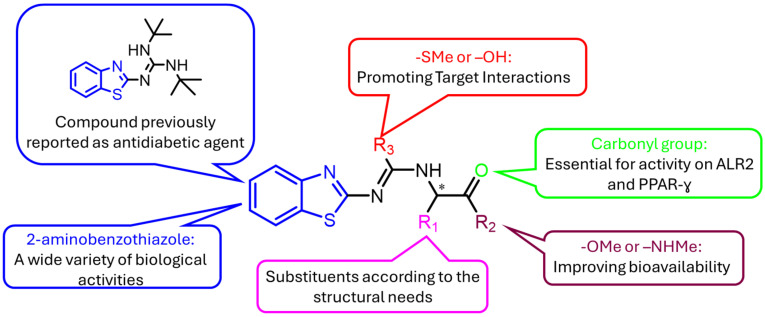
Rational design of 2-aminobenzothiazole derivatives evaluated. * Chiral carbon: structure with indefinite configuration.

Finally, the addition of isothiourea and urea groups was proposed to increase the number and intensity of interactions with the active sites of the targets, thus improving binding affinity. This general design concept is summarized schematically in Figure 1.

**Figure 2:**
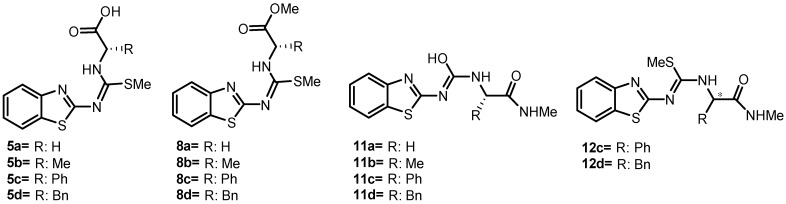
General chemical structure of 2-aminobenzothiazole derivatives linked to different isothioureas or ureas evaluated in silico. * Chiral carbon: structure with indefinite configuration.

Therefore, the present project aimed to synthesize and evaluate in silico, in vitro, and in vivo compounds derived from 2-aminobenzothiazoles (Figure 2) as ALR2 inhibitors, as well as to demonstrate their antihyperglycemic activity in a rat model with T2D induced by streptozotocin (STZ) and a high-fat diet (HFD) [15,24].

## 2. Results and Discussion

### 2.1. In Silico

Previously, the DIA-DB platform (https://bio-hpc.ucam.edu/dia-db/index.php, accessed on 28 December 2023) indicated that most of the benzothiazole derivatives from our chemical library have a high probability of interaction with the ALR2 enzyme, and in a minor proportion, with PPAR-γ, even if these structures do not possess the polar structural features described in the literature for such activity (carboxylic acids or hydantoin structure). For this reason, properties of sixty-four of these molecules were evaluated for oral administration, using specialized platforms, of which only the best fourteen are mentioned in this paper. This approach allows for early and efficient evaluation of the pharmacokinetic and toxicity profile, reducing the likelihood of compounds with unfavorable characteristics being present [15,18,25,26].

#### 2.1.1. Physicochemical Properties, Pharmacokinetic and Toxicologic Profile

Lipinski’s Rule of Five and other commonly used drug design filters were applied (Table 1). All compounds met the criteria for molecular weight, number of hydrogen bond donors and acceptors, solubility, and logP, indicating favorable oral bioavailability.

Medicinal chemistry filters, such as PAINS, Brenk, and lead-type filters, designed to identify structurally problematic, potentially reactive, or false positive compounds, were also used. No compounds generated relevant red flags, supporting their pharmacological viability [27].

**Table 1:** In silico physicochemical properties.

Physicochemical Properties										
	Lipinski’s Rule of Five						Aqueous Solubility			
	MW	cLogP	nON	nOHNH	nrot	TPSA	logS	[mg/mL]	Class	MR
**5d**	371.49	2.37	5	2	7	74.6	−5.09	3.05 × 10^−3^	III	104.71
**8c**	371.49	4.23	5	1	7	63.6	−5.11	2.92 × 10^−3^	III	104.22
**8d**	385.51	4.44	5	1	8	63.6	−5.29	1.97 × 10^−3^	III	109.03
**12d**	384.53	3.69	5	2	7	66.4	−4.93	4.55 × 10^−3^	III	110.75
**Zop**	419.38	3.54	6	1	5	85.1	−4.83	6.27 × 10^−3^	III	101.48
**Epa**	319.41	2.44	4	1	4	59.30	−4.19	2.08 × 10^−2^	II	91.76
**PGZ**	356.44	3.09	7	4	1	93.59	−4.31	1.76 × 10^−2^	III	102.59

Data obtained from Molinspiration (https://www.molinspiration.com, accessed on 2 January 2024) and ADMETLab 3.0 (https://admetlab3.scbdd.com/server/evaluationCal, accessed on 2 January 2024) [28,29]. MW, molecular weight; cLogP, octanol/water partition coefficient calculated; nON, number of hydrogen bond donors; nOHNH, number of hydrogen bond acceptors; nrot, number of rotal link donors; TPSA, topological polar surface area; MR, molar refractivity; Zop, zopolrestat; Epa, epalrestat; PGZ, pioglitazone.

Some inconsistencies were found regarding the prediction of blood–brain barrier (BBB) permeability; therefore, TPSA and cLogP values were used to create a boiled egg diagram, which was consistent with the behavior predicted by PreADMET (https://preadmet.webservice.bmdrc.org/, accessed on 2 January 2024) [27,30] and indicated the potential penetration of some isothioureas (**5d**, **8c**, **8d**) into the central nervous system.

Notably, several compounds in the five series possess ionizable carboxylic groups at physiological pH, which reduces their capacity for passive diffusion across lipophilic membranes, such as the BBB. This behavior is widely documented, as permeability depends both on the physicochemical properties of xenobiotics and on the participation of specific transporters or intracellular release strategies [31].

In this regard, most compounds exhibited high gastrointestinal absorption but limited permeability in cellular models (Table 2), with moderate permeability in Caco-2 and no permeability in MDCK, particularly for the tested compound **8d**, suggesting a low passive diffusion capacity. Oral and intestinal absorption predictions were consistent with lipophilicity values. Furthermore, the absence of interaction with P-glycoprotein (P-gp), the main efflux transporter, is advantageous, as it prevents active expulsion from the intestinal epithelium, promoting bioavailability and reducing the risk of drug resistance, a phenomenon particularly relevant in oncology [32,33].

One identified disadvantage was the inhibition of the OATP1B1 transporter, involved in hepatic drug uptake. This inhibition could reduce hepatic clearance and promote drug interactions. Likewise, the inhibition of the BSEP transporter, responsible for biliary excretion of bile acids, was predicted, which could lead to a risk of cholestasis or hepatotoxicity. In contrast, no inhibition of the BCRP transporter was observed, a favorable aspect since this transporter is involved in intestinal and renal elimination, and its inhibition is associated with systemic accumulation and toxicity [16,34].

**Table 2:** In silico pharmacological properties.

Pharmacokinetic									
	BBB	P-gp	CYP450 Inhibitor					%ABS	HIA
			1A2	2C19	2C9	2D6	3A4		
**5d**	√	×	√	√	√	×	×	83.27	98.24
**8c**	√	×	√	√	√	×	×	87.06	98.17
**8d**	√	×	√	√	√	×	×	87.06	98.06
**12d**	×	×	×	×	×	×	×	86.10	95.75
**Epa**	×	×	√	√	√	√	√	79.64	97.53
**Zop**	×	×	√	√	√	√	√	88.54	99.52
**PGZ**	×	×	√	√	√	√	√	76.71	97.35

Data obtained from PREADMET (https://preadmet.webservice.bmdrc.org/, accessed on 2 January 2024) and SwissADMET (https://www.swissadme.ch/, accessed on 3 January 2024) [30,35]. BBB, permeability of the blood–brain barrier; P-gp, P-glycoprotein substrate; CYP450, cytochrome P-450. ×, does not present; √, does present; %ABS, oral absorption percentage; HIA, human intestinal absorption.

Although the compounds could be subject to first-pass metabolism, none showed significant inhibition of the main cytochrome P450 (CYP450) isoforms, suggesting a minimal risk of drug interactions and acceptable metabolic stability [16,36,37].

Together, these characteristics favor a more stable and safer pharmacokinetic profile, with minimal interference in absorption, distribution, and elimination processes.

In this sense, the molecules evaluated do not present pharmacokinetic disadvantages, and in addition to this, it is suggested that none of the compounds is associated with toxic chronic effects (Table 3) belonging to class IV, indicating that the compounds could be harmful if ingested (300 < LD_50_ ≤ 2000 mg/Kg) according to the Globally Harmonized System (GHS) of Classification of Labeling of Chemicals [38].

#### 2.1.2. Molecular Docking

Considering that they have ideal properties, molecular docking was carried out against target molecules, both directed and blind, to ensure that the ligands bind to the orthosteric site (Table 4).

All the ligands tested interacted with the ALR2 active site pockets and have hydrophobic π–alkyl interactions with the cofactor, like the reference drugs and the literature reported [40]. Benzothiazole base is the main source of interaction with the cofactor and anion-binding pocket (ABP) residues by π–π, π–alkyl, and π–sulfur interactions.

**Table 4:** Molecular docking with ALR2 and PPAR-γ by Autodock4.

	ALR2 (4JIR *)				PPAR-γ (2FVJ **)			
	ΔG (Kcal/mol)	Ki (µM)	#IT	#I-ABP	ΔG (Kcal/mol)	Ki (µM)	#IT	#I-LBD
**5d**	−7.98	0.603	11	1	−8.45	0.338	18	6
**8b**	−7.29	1.770	8	0	−6.48	15.230	11	4
**8c**	−8.14	0.379	9	2	−8.80	0.122	19	0
**8d**	−8.39	0.262	13	1	−7.77	0.550	16	3
**11d**	−8.75	0.162	17	5	−7.65	1.140	18	8
**12d**	−8.62	0.171	16	3	−7.97	0.541	17	4
**Epa**	−8.50	0.359	23	7	−7.79	1.490	15	5
**Zop**	−8.40	0.328	19	9	−8.08	0.511	23	5
**PGZ**	−9.38	0.067	8	6	−9.48	0.099	11	4

* Crystal structure obtained from Protein Data Bank (RCSB PDB) (https://www.rcsb.org/structure/4JIR, accessed on 10 January 2024) [41]. ** Crystal structure obtained from RCSB PDB (https://www.rcsb.org/structure/2FVJ, accessed on 10 January 2024) [42]; ΔG = Gibbs free energy; Ki, inhibition constant; #IT, number of total interactions; #I-ABP, number of interactions with anion-binding pocket residues; #I-LBD, number of interactions with ligand-binding domain residues.

**Figure 3:**
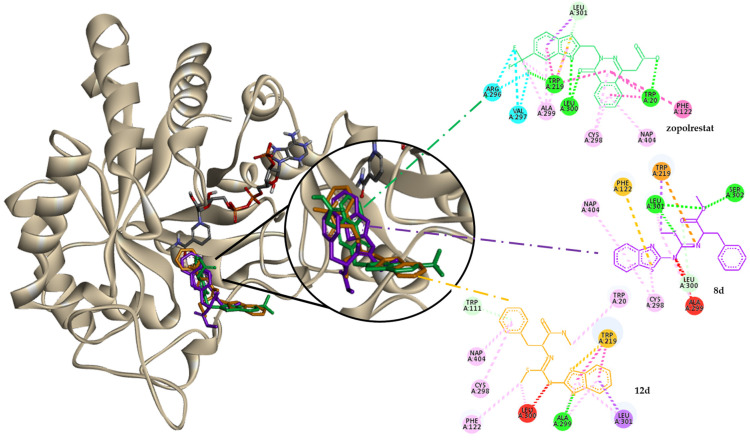
Behavior of molecules (zopolrestat, **8d,** and **12d**) in molecular docking with the ALR2 enzyme.

Despite the presence of carbonyl groups in the molecules evaluated, benzothiazole is the segment that remains in the ABP while its substituents enter the selectivity pocket with hydrogen interactions with Ala2999 and Leu300, contrary to what was observed with zopolrestat, so the interactions with His110 and Trp111 are π–π and not hydrogen bonds, as are usual with carboxylic acid inhibitors [43]; this behavior is represented in Figure 3.

Distance analysis allowed us to determine the preference of ligands for the selectivity pocket. However, they could still prevent the activation of ALR2 by glucose or lipoperoxidation products by preventing them from aligning to the catalytic site. The molecular volume of the mcompounds was close to 300 Å^3^, which is comparable to the reported for the inhibitors epalrestat and zopolrestat, and close to the estimated volume of the active site (398.8 Å^3^), according to the Proteins Plus Server (https://proteins.plus/, accessed on 12 January 2024) [44].

On the other hand, the ligands showed lower affinity for PPAR-γ, as measured by ΔG and Ki, but all were in the Y-shaped ligand-binding domain (LBD). Furthermore, the volume of the ligands is smaller than the cavity volume of this nuclear receptor, which is approximately 1200 Å^3^; nonetheless, they all present similar positions [22,45,46,47].

**Figure 4:**
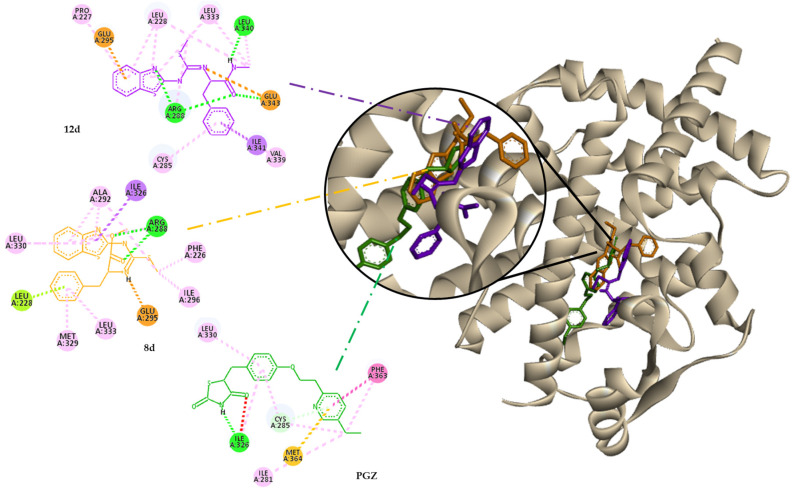
Behavior of molecules (PGZ, **8d,** and **12d**) in molecular docking with PPAR-γ.

There are different key residues in the LBD that induce receptor activation, depending on the type of agonist action, whether total or partial. In this case, molecules could be partial agonists because they interact with some important residues, such as Cys285, Arg288, and Ser289 of helix 3, like most of the partial agonists, which could promote the conformational change in helix 12, without a direct interaction with it. Although there were observed interactions with His449 of helix 10, which is not an essential residue for the direct activation of the receptor, it can stabilize the LBD cavity in some cases by positioning helix 12 in its closed and active conformation by indirect steric or electrostatic effects. This behavior is represented in Figure 4 [45,47].

It should be noted that, despite the hydrophobic nature of the active site, compounds presented an appropriate number of important interactions and well-affinity values due to the similarity of benzothiazole in the thiazolidine nucleus.

Considering their viability, attempts were made to optimize the reaction conditions of families C and D; however, the methods previously reported for synthesizing the amino acid derivatives proved to be non-reproducible, limiting the evaluation options (**8c**, **8d**, and **12d**).

### 2.2. Chemistry

The synthesis of the 2-aminobenzothiazole derivatives considered in this study is described in Figure 4. The key intermediate, compound **2**, was obtained by the reaction of 2-aminobenzothiazole with carbon disulfide in a strongly basic medium generated using aqueous KOH and DMF as solvent [48,49,50]. Modifications to the purification process in the reported procedure allowed the compound to be obtained as fine white crystals, unlike previous reports that describe it as a yellowish powder. This difference indicates a higher purity of the product, confirmed by High-Performance Liquid Chromatography (HPLC), where a single peak is observed at 7.6 min. This facilitated subsequent synthesis steps, especially for compounds with similar polarities.

**Figure 5:**
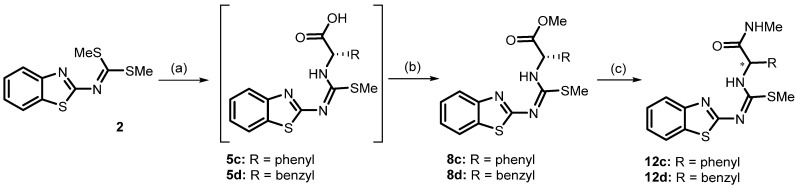
Synthetic procedure to obtain the 2-aminobenzothiazol derivatives: (**a**) 40% yield, TBAH 1.0 M in methanol/*L*-amino acid 1:1, room temperature, 24 h; (**b**) 62–80% yield, CH_3_I, DMF, 15 h; (**c**) 31% yield, 37% aqueous methylamine, MeOH and DMF, room temperature, 24 h. * Chiral carbon: structure with indefinite configuration.

This paper proposes a new synthetic method to obtain the previously described compounds **8c** and **8d**, in addition to reporting compound **12d** for the first time (Figure 5) [15,24]. The crude products of the described reactions were purified by column chromatography. Pure products were confirmed by Nuclear Magnetic Resonance (NMR) ^1^H and ^13^C and Infrared (IR) spectroscopy. Furthermore, high-resolution mass spectrometry (HRMS) and HPLC analyses using a chiral column were performed. Physicochemical properties, such as melting points, were also determined. Detailed information is provided in the Materials and Methods Section.

Compound **5d** was obtained according to the general procedure described [24]. It consists of reacting one molar equivalent of compound **2** with the corresponding carboxylate (L-phenylalanine sodium salt) under refluxing ethanol for 24 h. Under these conditions, the reaction proceeds by nucleophilic attack of the amino group on the carbonimidothioate group of compound **2**, with the elimination of gaseous thiomethanol to obtain the sodium salt of SMe-isothiourea **5d**, with a yield of 40%. For in vitro evaluation, it was proposed to obtain compound **5d** in its neutral form, which was not possible because it decomposes, even at low temperatures. Therefore, it was used only as an intermediate for the synthesis of its corresponding methyl ester (**8d**). The corresponding SMe-isothiureacarbozxylate methyl ester **8d** was obtained in 56% yield using one molar equivalent of the SMe-isothiurea sodium salt, with 1.2 equivalents of iodomethane in DMF at room temperature for 24 h, following the general procedure described in the literature [24]. Here, we report a modification to the protocol using tetrabutylammonium hydroxide (TBAH), a base soluble in both water and organic solvents. Its presentation in methanolic solution at known concentrations allowed precise control of the 1:1 stoichiometry with the amino acid, avoiding excess base that could induce undesired reactions in the benzothiazole nucleus. Thus, a *one-pot* reaction occurs via the equimolar reaction of L-phenylalanine, TBAH, and compound **2** at room temperature for 24 h, followed by the addition of 1.1 equivalents of iodomethane in DMF and an additional 15 h at room temperature, yielding compound **8d** in 62.1% yield. Compound **8c** was similarly prepared, obtaining a yield of 80.0% due to its lower degree of freedom. Previously reported X-ray diffraction confirmed the *E* configuration [24], while the obtention of an exclusive enantiomer of compounds **5d**, **8d**, and **8c** was confirmed by chiral HPLC.

Finally, compound **12d** was obtained by a nucleophilic substitution reaction at the carbonyl ester of compound **8d** during attempts to obtain urea **11d**. For this purpose, ester **8d** was treated with three equivalents of 37% aqueous methylamine in MeOH-DMF as solvent for 24 h at room temperature. It is noteworthy that, when attempting to carry out the reaction under reflux, as indicated in the methodology [24], or by prolonging the reaction time, complete hydrolysis was observed, resulting in the formation of 2-aminobenzothiazole as the main product.

The isolation of pure compound **12d** was confirmed by analysis of the ^1^H and ^13^C NMR spectra, which showed a single set of signals, and by HRMS, which showed an *m*/*z* value of 385.11408 for C_19_H_20_N_4_OS_2_, corresponding to **12d**. However, racemization of the compound during the synthesis process was confirmed by chiral HPLC, which showed two peaks with similar area ratios (HPLC 46:54). This racemization phenomenon is explained by the presence of a doubly activated acidic proton at its α-position of the carbonyl group and its α-position of the carbamimidothioate, which is removed due to, under these conditions, methylamine, which acts as both a nucleophile and a base, generating a planar enolate ion as intermediate. This intermediate is susceptible to reprotonation from both sides of the molecular plane, leading to the formation of both enantiomers.

### 2.3. In Vitro

Once the compounds were obtained (**8c**, **8d**, and **12d** as a racemic mixture), they were evaluated in vitro as potential inhibitors of the ALR2 enzyme, using epalrestat as the reference drug. Furthermore, their anti-radical and antioxidant capacities were analyzed to determine whether they could exert a protective effect through an intrinsic antioxidant mechanism of the compounds in the body.

#### 2.3.1. Inhibitory Activity Against ALR2

**Figure 6:**
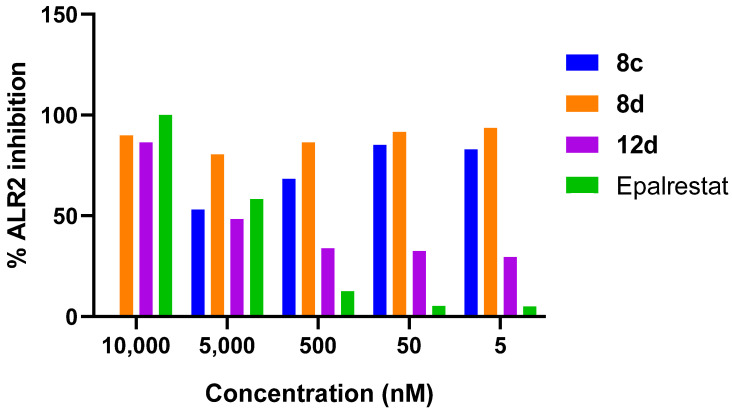
ALR2 inhibition activity as a function of concentration of 2-aminobenzothiazole derivatives (**8c**, **8d**, and **12d** as a racemic mixture).

The tested compounds demonstrate enhanced ALR2 inhibitory activity compared to epalrestat at all concentrations tested (Figure 6). This observation is supported by the results of the molecular docking analysis, where derivatives **8c**, **8d**, and **12d** displayed key interactions like those of the reference drug, including hydrogen bond formation with the selectivity pocket and π–alkyl interactions with ABP residues. However, despite their inhibitory capacity, compounds **8c** and **8d** did not show a concentration-dependent response, preventing accurate calculation of their IC_50_ values. This behavior suggests that they might act via an atypical or mixed inhibition mechanism, warranting more detailed kinetic studies. Notably, the blind docking predicted a high affinity of these compounds for the orthosteric site of the enzyme but did not rule out possible allosteric interactions or multiple binding modes, opening new lines of investigation into their mechanism of action.

Inhibition of the ALR2 enzyme could contribute to the reduction in systemic oxidative stress; however, in an in vivo model, it is difficult to attribute this reduction to the inhibition of this enzyme directly. Therefore, the intrinsic antioxidant capacity of the compounds was also evaluated using two complementary assays: ferric reducing antioxidant power (FRAP) and the 2,2′-azinobis-(3-ethylbenzothiazoline-6-sulfonic acid) radical cation assay (ABTS) [51].

#### 2.3.2. Antioxidant Capacity

First, the FRAP assay revealed low antioxidant capacity for compounds. Overall, the compounds managed to reduce less than 20% of the ferric ion, even at high concentrations, which rules out a significant antioxidant action through this pathway (Table S7).

On the other hand, the results obtained in the ABTS assay were unexpected. Normally, the reduction in the ABTS radical cation is manifested by a decrease in absorbance; however, in the presence of the tested compounds, an increase in the absorbance of the reaction mixture was observed. This phenomenon cannot be attributed to the intrinsic absorption of the compounds, as they did not present a signal at the wavelength used in the assay. As a result, negative percentage reduction values were obtained for the ABTS cation.

A hypothesis, not yet verified, was proposed, suggesting the possible formation of a cationic complex between the compounds and ABTS, which could explain this anomalous behavior. This observation opens new lines of research, as a similar phenomenon had not been previously described. It is noteworthy that the method was successfully validated using 5-aminosalicylic acid (5-ASA) as a positive control.

Considering these and based on the correlation reported between the ABTS and FRAP methods in the literature [51], it is concluded that the analyzed compounds do not exhibit significant intrinsic antioxidant capacity.

### 2.4. In Vivo

#### 2.4.1. Acute Oral Toxicity (AOT)

All synthesized compounds (**8c**, **8d**, and **12d** as a racemic mixture) were suspended in 2% Tween 80 in water and administered orally by an esophageal cannula following the “Up and Down” methodology. Nevertheless, compounds could not be solubilized in the desired volume to complete the highest dose (1750 mg/Kg), so the toxicity test was performed with a maximum dose of 1250 mg/Kg. Even so, the values obtained indicate that these compounds are classified within toxicity category 4 according to the guidelines established by the Organization for Economic Co-operation and Development (OECD) [52].

Animals did not present any apparent adverse effects at subsequent hours after administration. After 14 days, their weight gains and organ size did not present a significant difference from a rat without treatment (Table 5). This leads us to conclude that the products evaluated are safe.

#### 2.4.2. Antidiabetic Activity

HFD replicates an initial insulin resistance state [53], and STZ administration generates a cachexia state due to muscle wasting, lipolysis, and proteolysis associated with T2D induction [54,55]. It is worth mentioning that during the present project, the T2D induced was only 40% successful, so the “*n*” had to be reduced to four animals per group [56,57]. It should be noted that in previous studies, we demonstrated that vehicle administration did not cause significant changes in any metabolic parameters, so this group was not considered for this evaluation [15].

##### Acute Evaluation

**Figure 7:**
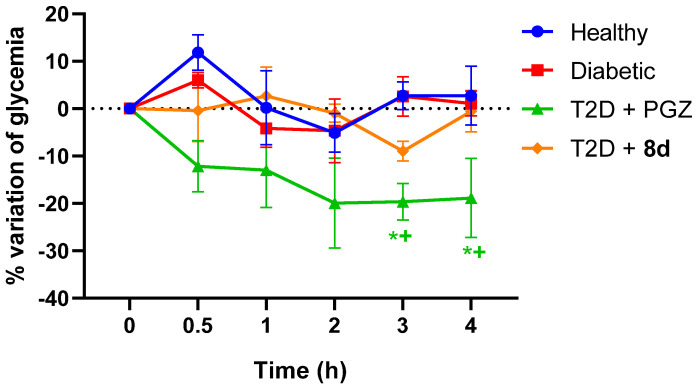
Effect on blood glucose levels after a single administration of treatments in the STZ-HFD-induced T2D model, without prior fasting. Data reported as the mean ± SEM with *n* = 4. * Significant difference against healthy control (*p* < 0.05); + significant difference against diabetic control (*p* < 0.05).

This evaluation is based on the ability of the drug or compound to lower blood glucose under normal conditions, without a previous fast [15]. There was no notable downward slope in glycemic values in any treated group. Even after normalizing values, the behavior of the healthy, diabetic, and T2D + **8d** groups did not show significant differences. Furthermore, **8d** kept values lower than PGZ and stable despite food consumption (~200 mg/dL) (Figure 7). This suggests that there is no risk of hypoglycemia.

The behavior mentioned in both acute and subchronic models could be due to the mechanism of the different treatments since, in the known case of PGZ, by activating PPAR-γ, it regulates the transcription of genes related to glucose and lipid metabolism, which takes time and may not translate into an immediate decrease in glycemia [58,59,60,61].

##### Subchronic Evaluation

**Figure 8:**
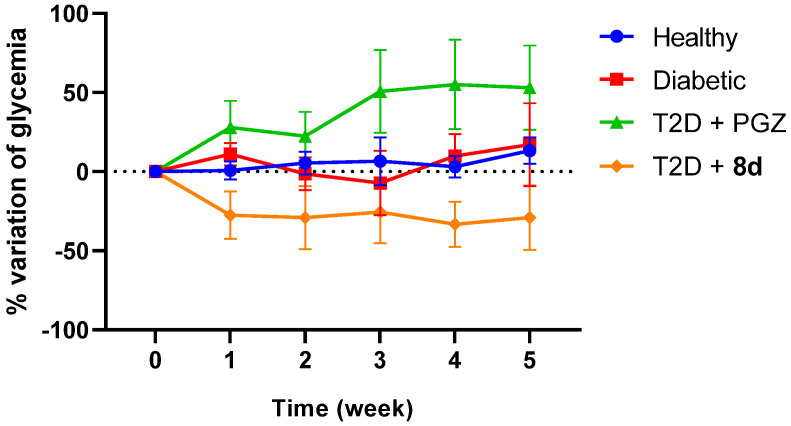
Effect of treatments on fasting blood glucose levels through subchronic assessment. Data represented as the mean ± SEM with an *n* = 4.

The daily administration of the treatment for **8d** allowed for maintaining lower blood glucose levels close to the normoglycemia of the healthy group. Even when normalizing the values (Figure 8), it is notable that this treatment is the one that lowers blood glucose during the 5 weeks of treatment.

**Figure 9:**
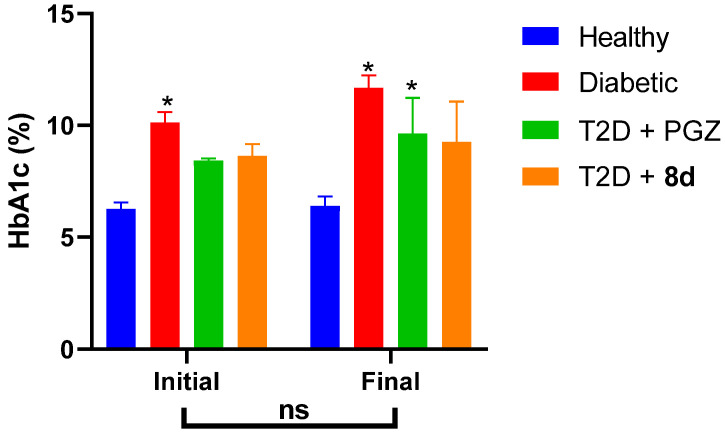
Effect on glycated hemoglobin percentage after treatment period. Comparative bar graph of HbA1c values at the beginning and end of the respective treatments. Data represented as the mean ± SEM with an *n* = 4. * Significant difference against healthy control (*p* < 0.05); ns, no significant difference (*p* > 0.05).

This trend was consistent with the glycated hemoglobin (HbA1c) levels obtained at the end of treatment, as it limited the formation of advanced glycation end products (AGEs), just as PGZ did, even though, with this reference drug, the animals reached levels like diabetic control after 2 weeks of treatment (Figure 9). Furthermore, the decrease in the HOMA-IR index in these two treated groups evidences an improvement in insulin sensitivity, which was more significant in the T2D + **8d** group, suggesting a possible effect on peripheral insulin resistance (Table 6) [53]. Furthermore, an oral glucose tolerance test (OGTT) was shown to improve insulin sensitivity in treated groups, which reduced both the area under the curve and the time to return to baseline glycemic levels (Figure S27) [62].

**Figure 10:**
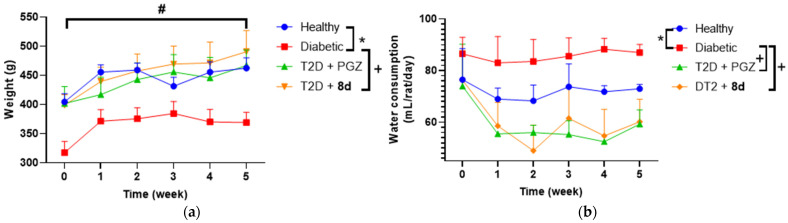
Monitoring. (**a**) Weight of the rats during the progress of the treatment weeks and (**b**) water consumption per rat per day during the progress of the treatment weeks of the different study groups. Data represented as the mean ± SEM with *n* = 4. * Significant difference against healthy control (*p* < 0.05); + significant difference against diabetic control (*p* < 0.05); # significant difference against the initial value (*p* < 0.05).

All these results could be reflected in the weight and water consumption of the distinct groups (Figure 10). T2D + PGZ and T2D + **8d** had similar behaviors, decreasing the polydipsia after the first week of treatment.

To evaluate the impact of the treatments on other metabolic parameters, blood chemistries of 24 elements were performed [59,63].

**Figure 11:**
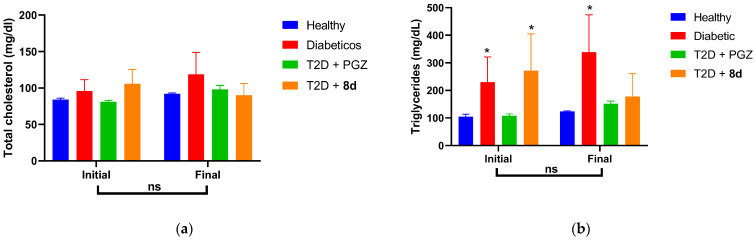
T-chol and TG values. Comparative bar graph of the values of (**a**) T-chol and (**b**) TGs at the beginning and end of the respective treatments. Data represented as the mean ± SEM with *n* = 4. * Significant difference against healthy control (*p* < 0.05). ns, no significant difference (*p* > 0.05).

Figure 11 shows that the induction of T2D did not significantly alter total cholesterol (T-chol), but it did increase triglycerides (TGs), which were controlled after 5 weeks of treatment. Compound **8d** was notable for significantly reducing TG [64].

Alanine aminotransferase (ALT), aspartate aminotransferase (AST), gamma-glutamyltransferase (GGT), and alkaline phosphatase (ALP) levels did not show significant differences compared to the healthy group, suggesting the absence of hepatotoxicity. All of them are often used to determine hepatotoxicity; nevertheless, while ALT is the most selective of liver cells, AST is also found in the heart, muscle, and kidneys (Figure 11). Therefore, the fact that the ALT values of the T2D + **8d** group were like those of the T2D + PGZ group indicates potential hepatoprotective activity [65,66].

**Figure 12:**
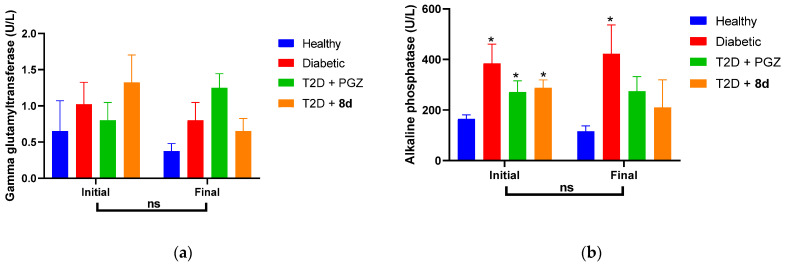
Parameters of the liver profile. Values of (**a**) GGT and (**b**) ALP of groups at the beginning and end of the experimentation time. Data represented as the mean ± SEM with *n* = 4. * Significant difference vs. healthy control (*p* < 0.05). ns, no significant difference (*p* > 0.05).

On the other hand, GGT is located on the external surface of liver and biliary cells, where it facilitates the transport of glutathione into the cell interior. Therefore, it is considered a sensitive marker of oxidative stress, insulin resistance, and various cardiovascular risk factors [67]. In this context, the decrease in GGT levels observed in some groups at the end of treatment, together with ALP values, suggests that the compounds evaluated do not induce liver toxicity. The behavior of the group treated for **8d** could be related to a possible hepatoprotective effect and an improvement in metabolic status (Figure 12) [64,65,66].

ALP is an enzyme expressed in the liver and biliary tract, and its levels can be altered by hepatic inflammatory processes or biliary dysfunction, conditions that can present as complications of diabetes mellitus. In this way, the development of cholestasis and fatty liver is prevented [67]. Furthermore, like GGT, ALP participates in antioxidant defense by stimulating extracellular glutathione transport [68]. Therefore, the simultaneous reduction in GGT and ALP levels observed after administration of **8d** suggests a possible antioxidant mechanism mediated by enzyme modulation. This is particularly relevant given that, as mentioned above, the compounds do not exhibit intrinsic anti-radical activity [64,65,67,68,69].

### 2.5. Ex Vivo

#### 2.5.1. Determination of the Antioxidant Effect

In this study, total antioxidant capacity (TAC) levels decreased significantly in the untreated diabetic group, confirming a state of systemic oxidative stress (Table 7) [70].

Treatment with PGZ promoted a partial recovery of TAC, without reaching the levels of the healthy group, which is supported by the activation of the AMP-activated protein kinase—the Glutaminase 1 (AMPK-GLS1) axis described in the literature [71]. In contrast, compound **8d** did not improve TAC and even showed a further reduction, indicating the absence of an antioxidant effect under the conditions evaluated. However, this result does not rule out a possible indirect modulation of antioxidant enzymes, which should be explored in future studies.

#### 2.5.2. Quantification of Inflammation Markers

Chronic low-grade inflammation, common in obesity and DM, is mediated by cytokines, such as IL-6 and TNF-α, produced by adipocytes, and associated with endothelial dysfunction and cardiovascular complications [64].

In this study, PGZ significantly reduced IL-6 and TNF-α levels, confirming its anti-inflammatory effect. In contrast, compound **8d** presented the highest levels of both cytokines, suggesting a possible inflammatory exacerbation. However, considering the positive impact of compounds on glycemic levels, this could be primarily due to the adipogenic effect and significant weight gain compared to the control groups (Table 8 and Table 9).

These results correlate with observed levels of oxidative stress, with R^2^ coefficients of 0.98 for TNF-α, supporting the relationship between oxidative stress and inflammatory dysregulation [72]. Furthermore, increased creatine kinase (CK) levels suggest a possible component of cardiac damage, which could also be related to the observed inflammatory profile [73].

**Figure 13:**
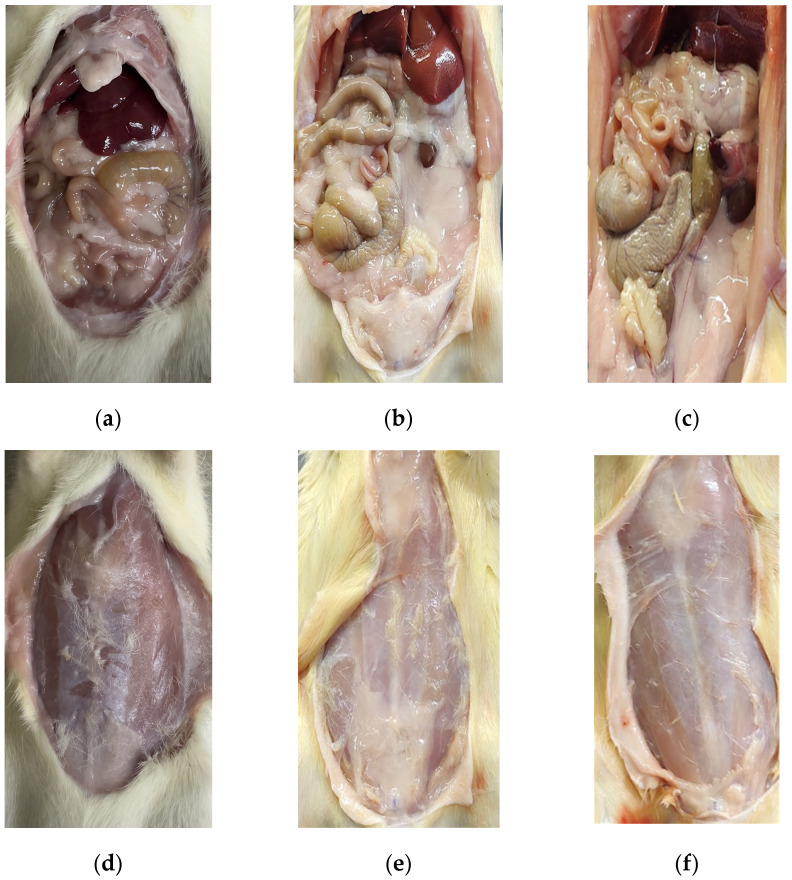
Representative images obtained from the necropsy of animals: Adipogenic effect in (**a**) healthy, (**b**) PGZ, and (**c**) **8d**; (**d**) necropsy of a healthy rat; and subcutaneous adipose tissue redistributed by the action of treatments (**e**) PGZ and (**f**) **8d**.

Finally, the necropsy findings confirmed a clear adipogenic effect following treatment with PGZ and **8d**, accompanied by an evident redistribution of adipose tissue from visceral to subcutaneous fat. This redistribution was marked in the T2D + PGZ group, whereas in the T2D + **8d** group, the effect was barely perceptible. This suggests that a longer treatment period may be necessary for **8d** to induce comparable changes. Activation of PPAR-γ is known to stimulate adipogenesis in subcutaneous depots, increasing the number of small adipocytes and promoting lipid storage in metabolically benign regions. This process contributes to improved insulin sensitivity and reduced lipotoxicity associated with visceral adipose tissue. Thus, the mechanism observed with PGZ would explain the adipose redistribution and suggests a similar, albeit less pronounced, effect with **8d** under extended treatment conditions (Figure 13) [4,74,75].

Taken together, our results strongly suggest that compound **8d** possesses the ability to inhibit ALR2 both in vitro and in vivo, as recent studies have shown that inhibition of this enzyme can reduce hepatic lipid accumulation and improve hyperglycemia-induced dyslipidemia. These effects are attributed to the activation of PPAR-α, which promotes lipid degradation and suppresses fructose-mediated lipogenesis. However, in our model, compound **8d** also showed high affinity for PPAR-γ, improving insulin sensitivity and exerting an adipogenic effect. These findings open the possibility that this 2-aminobenzothiazole derivative acts as a multitargeted agent, simultaneously modulating ALR2 and PPAR-α/γ receptors, which could increase its therapeutic efficacy in the treatment of metabolic disorders associated with T2D [20].

## 3. Materials and Methods

### 3.1. In Silico

#### 3.1.1. Prediction of Physicochemical Properties, Pharmacokinetic and Toxicological Profile

The molecules’ smile codes were submitted at online platforms such as Molinspiration Cheminformatics (https://www.molinspiration.com, accessed on 2 January 2024), SwissADMET (https://www.swissadme.ch/, accessed on 3 January 2024), PreADMET/Tox (https://preadmet.webservice.bmdrc.org/, accessed on 2 January 2024), ProTox-II (https://tox.charite.de/protox3/, accessed on 6 January 2024), and DIA-DB (https://bio-hpc.ucam.edu/dia-db/index.php, accessed on 28 December 2023) to predict physicochemical, pharmacological, and toxicological properties [25,28,29,30,35].

#### 3.1.2. Molecular Docking

Molecules were drawn with ChemDraw 18.1, optimized on Avogadro using a semiempirical force file (MMFF94), and prepared by torsion tree in AutodockTools 1.5.7.

The crystal structures of ALR2 and PPAR-γ were obtained from PDB (ID: 4JIR and 2FVJ), which were prepared on Chimera 1.15, where just crystal water was removed, preserving the NADPH and coactivator nuclear receptor 1 structures, respectively.

Polar hydrogens, such as Kollmann partial loads, were added to the enzyme, and grid boxes were established considering the active sites: 60 × 60 × 60 Å cubic mesh and a mesh spacing of 0.375 Å with the coordinates X = −7.694, Y = 5.653, and Z = 18.187 for ALR2 docking [41] and X = 0.532, Y = 22.616, and Z = 21.372 for PPAR-γ [42]. The cubic mesh was extended to 126 × 126 × 126 Å using the same coordinates as in the blind coupling case. Docking was performed by genetic and Lamarckian algorithms with 100 runs, a population size of 100, 10,000,000 as a maximum number of evaluations, and 100 generations for picking the worst individuals.

The validation was confirmed by redocking the co-crystallized ligand, and the RMSD was shown to be no more than 1.5 Å. The binding free energy (∆G) of the most populated cluster and representative pose was analyzed by visualization of the interaction with the active site using BIOVIA Discovery Studio 2020 [76].

### 3.2. Chemistry

The reagents purchased from Sigma-Aldrich (St. Louis, MO, USA) were used without further purification. The progress of the reactions was routinely monitored by thin-layer chromatography (TLC) on silica gel 60 (precoated Merck F254 plates, Darmstadt, Germany) under UV lamp irradiation (254 nm). Melting points were measured on a Melt-Temp “Electrothermal” (American Laboratory Trading, San Diego, CA, USA) and were uncorrected. Reactions were concentrated on a standard rotary evaporator (Buchi Labortechnik AG, Flawil, Switzerland) and purified by column chromatography on silica gel (230–400 mesh).

^1^H and ^13^C NMR were recorded on a Bruker ASCEND 400 spectrometer (Bruker BioSpin, Billerica, MA, USA) at 400 and 101 MHz, respectively, using deuterated chloroform and tetramethyl silane as internal standard. The data will be reported as follows: chemical displacement in ppm (δ), integration area, multiplicity (s = singlet, s br = wide singlet, d = doublet, t = triplet, q = quartet, and m = multiplet), and coupling constants in Hz (*J*). The IR spectra were obtained on Perkin-Elmer (PC16 Spectrum GX and Frontier) FT-IR spectrometers with attenuated total reflectance (ATR) accessories (Perkin-Elmer, Waltham, MA, USA).Finally, the presence of the molecular ion of each compound will be corroborated in a mass spectrometer using the electrospray ionization mode. Direct mass spectrometry analysis to determine low-resolution spectra (DART+) was performed on a Bruker micrOTOF-Q instrument (Bruker, Billerica, MA, USA), and electrospray ionization high-resolution mass spectrometry (ESI+) was performed on a Bruker Daltonics micrOTOF-Q instrument (Bruker Daltonics Corporation, Billerica, MA, USA).

The HPLC analysis was conducted in a Waters HPLC Alliance 2695 coupled to UV/visible detector and a diode array Waters 2996 (Marshall Scientific, Hampton, NH, USA) and the following separation conditions: column Chiralpak AD-H; 92:8 hexane-IPA, and Flux 1.0 mL/min.

#### 3.2.1. Synthesis of the Dimethyl Benzo[*d*]thiazol-2-ylcarbonimidodithioate Intermediate [Compound **2**]

A 1 L flat-bottomed flask on an ice bath 2-aminobenzothiazole (7.51 g, 50 mmol) was dissolved in 50 mL of DMF; then, 3 mL of a 20 M NaOH solution was added drop by drop, and once the formation of a gray precipitate was observed, it was allowed to react for 30 min before slowly adding CS_2_ (6 mL, 100 mmol). After 30 min, 3 mL of 20 M NaOH was added again and allowed to react for 60 min. Finally, CH_3_I (5.7 mL, 100 mmol) was added, and after approximately 5 min, the formation of a bright yellow precipitate was observed. The formed solid was washed with 900 mL of cold distilled water in constant agitation and then filtered and washed with cold water until the excess base was removed. The resulting solid was purified by column chromatography (Hex-AcOEt 97:3).

Compound **2** was obtained with a yield of 66.4% (8.43 g, 33.2 mmol) and an mp = 70–72 °C (lit. [49,77], mp = 72–73 °C); **TLC** R_f_ = 0.34 (Hex-AcOEt 9:1); **HPLC** t_r_ = 7.6 min; **^1^H NMR** (400 MHz, CDCl_3_) δ (ppm): 7.91 (1H, ddd, *J* = 8.1, 1.3, 0.6 Hz, H-4), 7.77 (1H, ddd, *J* = 7.9, 1.4, 0.6 Hz, H-7), 7.42 (1H, ddd, *J* = 8.3, 7.3, 1.3 Hz, H-5), 7.29 (1H, ddd, *J* = 8.3, 7.3, 1.1 Hz, H-6), 2.62 (6H, s, SCH_3_); **^13^C NMR** (101 MHz, CDCl_3_) δ (ppm): 174.58 (C, N=C(SCH_3_)_2_), 167.38 (C, C2), 151.38 (C, C3a), 134.62 (C, C7a), 125.91 (CH, C5), 124.08 (CH, C6), 122.33 (CH, C7), 121.19 (CH, C4), 15.8 (2CH_3_, SCH_3_); **ATR-FTIR** ν_max_ (cm^−1^): 3076.8, 3053.4, 2983.1, 2916.9; **SM (DART+)**: 123(15), 149(20), 151(15), 153(10), 255 (100) [M + H]^+^, 256(10).

#### 3.2.2. Synthesis of (*E*)-((benzo[*d*]thiazol-2-ylimino)(methylthio)methyl)-L-phenylalanine [**5d**]

In a flask, L-phenylalanine (202 mg, 1.23 mmol) and NaOH (48.8 mg, 1.2 mmol) were placed in 10 mL of EtOH. This reaction mixture was allowed to react for 1 h at 50 °C in constant agitation, and as soon as the homogeneous solution was observed, compound **2** (311.7 mg, 1.2 mmol) was added. It was kept at the same temperature for 24 h, and the reaction was monitored with TLC (Hex-AcOEt 7:3). It was neutralized at pH 6 and purified by column chromatography (9:1). Compound **5d** was obtained as an acid in the form of a pale white crystal with a yield of 40.0% (182.5 mg, 0.5 mmol) and an mp = 92−94 °C; **TLC** R_f_ = 0.44 (Hex-AcOEt 7:3); **HPLC** t_r_ = 10.7 min; **ATR-FTIR** ν_max_ (cm^−1^): 3155.8, 3067.3, 2925, 2855.6; **HRMS (ESI+)** 372.0840 *m/z* calculated for C_18_H_18_N_3_O_2_S_2_; found 372.0928 [M + H]^+^. The compound decomposes easily under ambient conditions, so it was not possible to characterize it by NMR.

#### 3.2.3. Synthesis of Methyl (*E*)-((benzo[*d*]thiazol-2-ylimino)(methylthio)methyl)-L-phenylalaninate [**8d**]

In a flask provided with a magnetic stirrer, L-phenylalanine (1.65 g, 10 mmol) and TBAH (10 mL of 1.0 M, 10 mmol) were placed, allowing them to react for 15 min at room temperature, until completely dissolved. Then, compound **2** (2.54 g, 10 mmol) was added. After 24 h of reaction, 10 mL of DMF was added and, in the ice bath, CH_3_I (0.75 mL, 11.6 mmol) was aggregated. Immediately after addition, the flask was sealed to prevent the reactant from volatilizing, and it was kept in constant agitation at room temperature for 15 h. The product was then isolated by extraction with AcOEt (3 × 15 mL) and purified by column chromatography (Hex-AcOEt 9:1). Compound **8d** was obtained with a yield of 62.1% (2.4 g, 6.2 mmol) and an mp = 83–84 °C (lit. [24]; mp = 84–85 °C); **TLC** R_f_ = 0.45 (Hex-AcOEt 8:2); **HPLC** t_r_ = 6.4 min; **^1^H NMR** (400 MHz, CDCl_3_) δ (ppm): 11.16 (1H, s br, NH), 7.64 (1H, ddd, *J* = 7.9, 1.4, 0.6 Hz, H-4), 7.60 (1H, d, *J* = 8.0 Hz, H-7), 7.31 (1H, ddd, *J* = 8.2, 7.3, 1.3 Hz, H-5), 7.31–7.17 (5H, m, H-6 and H-Ph), 7.17 (1H, ddd, *J* = 7.9, 7.3, 1.2 Hz, H*_para_*-Ph), 4.65 (1H, d br, *J* = 7.5 Hz, CHCO_2_CH_3_), 3.70 (3H, s, OCH_3_), 3.21 (2H, d, *J* = 6.2 Hz, CH_2_Ph), 2.46 (3H, s, SCH_3_); **^13^C NMR** (101 MHz, CDCl_3_) δ (ppm): 171.45 (C, C2), 171.01 (C, CO_2_CH_3_), 164.15 (C, NCSCH_3_), 151.03 (C, C3a), 135.35 (C, benzyl), 132.25 (C, C7a), 129.42 (2CH*_ortho_*, benzyl), 128.65 (2CH*_meta_*, benzyl), 127.31 (CH, C5), 125.51 (CH*_para_*, benzyl), 123.24 (CH, C6), 120.96 (CH, C7), 120.61 (CH, C4), 58.18 (CH, CHCO_2_CH_3_), 52.52 (CH_3_, OCH_3_), 39.16 (CH_2_, CH_2_Ph), 14.09 (CH_3_, SCH_3_); **ATR-FTIR** ν_max_ (cm^−1^): 3155.8, 3059.8, 3028.8, 2971.2, 2925; **SM (DART+)**: 386 (55) [M + H]+, 387(13), 386(8); **HRMS (ESI+)** 386.0997 *m/z* calculated for C_19_H_20_N_3_O_2_S_2_; found 386.0991 [M + H]^+^.

#### 3.2.4. Synthesis of Methyl (*RS, E*)-*N*’-(benzo[*d*]thiazol-2-yl)-*N*-(1-(methylamino)-1-oxo-3-phenylpropan-2-yl)carbamimidothioate [**12d**]

Compound **8d** (770 mg, 2.0 mmol) was dissolved in 15 mL of MeOH and 5 mL of DMF. The mixture was allowed to react with 37% aqueous methylamine (0.5 mL, 6.0 mmol) at room temperature for 24 h. Compound **12d** was purified by column chromatography (Hex-AcOEt 8:2). Compound **12d** was obtained as a racemic mixture and with a colorful solid appearance with a yield of 31% (238.1 mg, 0.6 mmol) and an mp = 188-189 °C; **TLC** R_f_ = 0.31 (Hex-AcOEt 7:3); **tr** = 9.8 and 10.76 min (46:54); **^1^H NMR** (400 MHz, CDCl_3_) δ (ppm): 10.89 (1H, s br, NH), 7.64 (1H, dd, *J* = 7.8, 1.3 Hz, H-4), 7.57 (1H, d, *J* = 8.1 Hz, H-7), 7.33 (1H, ddd, *J* = 8.2, 7.3, 1.3 Hz, H-5), 7.30–7.14 (6H, m, H-6 and H-Ph), 5.92 (1H, s br, NHMe), 4.49 (1H, d br, *J* = 8.2 Hz, CHCONHMe), 3.30 (1H, dd, *J* = 13.9, 6.4 Hz, CHHPh diastereotopic proton), 3.17 (1H, dd, *J* = 13.9, 5.3 Hz, CHHPh diastereotopic proton), 2.73 (3H, d, *J* = 4.9 Hz, NHCH_3_), 2.44 (3H, s, SCH_3_); **^13^C NMR** (101 MHz, CDCl_3_) δ (ppm): 170.58 (C, C2), 135.80 (C, benzyl), 132.22 (C, C7a), 129.66 (2CH*_ortho_*, benzyl), 128.70 (2CH*_meta_*, benzyl), 127.28 (CH, C5), 125.70 (CH*_para_*, benzyl), 123.52 (CH, 6C), 121.09 (CH, C7), 120.58 (CH, C4), 60.0 (CH, CHCONHCH_3_), 38.81 (CH_2_, CH_2_Ph), 26.36 (CH_3_, NHCH_3_), 14.17 (CH_3_, SCH_3_); **ATR-FTIR** ν_max_ (cm^−1^): 3290.2, 1738.6, 1661.9; **SM (DART+)**: 337(8), 368(10), 385(55) [M + H]^+^, 386(12), 387(6); **HRMS (ESI+)** 385.11157 *m/z* calculated for C_19_H_20_N_4_OS_2_; found 385.11408 [M + H]^+^.

#### 3.2.5. Synthesis of Methyl (*S,E*)-2-(((benzo[*d*]thiazol-2-ylimino)(methylthio)methyl)amino)-2-phenylacetate [**8c**]

In a flask provided with a magnetic stirrer, L-phenylglycine (1.65 g, 10.0 mmol) and TBAH (10 mL of 1.0 M, 10 mmol) were placed, allowing them to react for 10 min at room temperature, until they were completely dissolved. Then, compound **2** (2.54 g, 10 mmol) was added. After 24 h of reaction, 10 mL of DMF was added, and, in the ice bath, CH_3_I (0.75 mL, 11.6 mmol) was aggregated. Immediately after addition, the flask was sealed to prevent volatilization of the reagent, and it was kept in constant agitation at room temperature for 15 h. The product was then isolated by extraction with AcOEt (3 × 15 mL) and purified in column chromatography (Hex-AcOEt 9:1). Compound **8c** was obtained with a yield of 80.0% (2.97 g, 8.0 mmol) and an mp = 180–181 °C; **TLC** R_f_ = 0.475 (Hex-AcOEt 8:2); **tr** = 6.3; **^1^H NMR** (400 MHz, CDCl_3_) δ (ppm): 11.86 (1H, s br, NH), 7.62 (1H, ddd, *J* = 8.2, 1.2, 0.6 Hz, H-4), 7.55 (1H, ddd, *J* = 7.9, 1.4, 0.6 Hz, H-7), 7.37–7.32 (2H, m, H-5 and H-6), 7.27–7.19 (4H, m, H-Ph), 7.11–7.04 (1H, m, H*_para_*-Ph), 5.30 (1H, d, *J* = 3.0 Hz, CHCO_2_CH_3_), 3.63 (3H, s, OCH_3_), 2.33 (3H, s, SCH_3_); **^13^C NMR** (101 MHz, CDCl_3_) δ (ppm): 171.67 (C, C2), 170.10 (C, CO_2_CH_3_), 163.95 (C, NCSCH_3_), 151.07 (C, C3a), 136.08 (C, benzyl), 132.21 (C, C7a), 128.98 (2CH*_meta_*, benzyl), 128.74 (CH*_para_*, benzyl), 127.20 (2CH*_ortho_*, benzyl), 125.58 (CH, C5), 123.32 (CH, C6), 120.97 (CH, C7), 120.78 (CH, C4), 60.56 (CH, CHCO_2_CH_3_), 53.11 (CH_3_, OCH_3_), 14.12 (CH_3_, SCH_3_); **ATR-FTIR** ν_max_ (cm^−1^): 2972.1, 1739.8, 1434.3; **SM (DART+)**: 374(4), 373(8), 372(100) [M + H]^+^; **HRMS (ESI+)** 372.0840 *m/z* calculated for C_18_H_17_N_3_O_2_S_2_; found 372.0849 [M + H]^+^.

### 3.3. In Vitro

#### 3.3.1. Inhibitory Activity Against ALR2

Enzyme inhibitory assays for the synthesized compounds (**8c**, **8d**, and **12d** as a racemic mixture) were carried out in triplicate using the ALR2 activity assay kit (ab273276) according to the manufacturer’s instructions [78]. The obtained dose–response curve had concentrations of 10 μM, 5 μM, 500 nM, 50 nM, and 5 nM.

#### 3.3.2. Evaluation of Antioxidant Capacity

All synthesized compounds were evaluated at 30 μM, 50 μM, 70 μM, 90 μM, and 110 μM under two of the most widely used methods in research to directly measure the antioxidant and anti-radical intrinsic capacity, FRAP, and ABTS assay, which were validated by a dose–response curve of 5-ASA as a reference [50]. In the same way, these tests were carried out in triplicate using an assay kit (from Pharmaceutical and Biotechnological Innovation-Services) according to the manufacturer’s instructions [79].

### 3.4. In Vivo

Male Wistar rats (200 ± 20 g) were acquired from the bioterium of the Instituto de Ciencias de la Salud (ICSA), Universidad Autónoma del Estado de Hidalgo.

The study complies with the Mexican norm for this matter (NOM-062-ZOO-1999, Technical Specifications for the Production, Care, and Use of Laboratory Animals, SAGARPA), as well as the Guide for the Care and Use of Laboratory Animals of the National Research Council and National Institutes of Health (NIH Publication No. 8023, revised 1978).

#### 3.4.1. Acute Oral Toxicity

The “Up and Down” procedure of OECD Guideline 425 was used to estimate the LD_50_ value of synthesized compounds. Briefly, a dose of 175 mg/Kg was administered to a male Wistar rat, which was monitored continuously for 48 h. As the animal survived, a second animal received a dose of 550 mg/Kg, and, as it survived, a third animal received an increasing dose of 1750 mg/Kg. Finally, two other rats were administered the same dose. All animals were kept under observation for 14 days, and 24 h later, they were taken to the humane endpoint for macroscopic examination of organs and tissues. Finally, the LD_50_ value of the compounds was estimated to determine their degree of toxicity and classified based on the GHS of Classification and Labeling of Chemicals [38,52].

#### 3.4.2. Antidiabetic Activity

In vivo experiments were conducted in an acute and a subchronic model, where Wistar rats (180–220 g) were used. The study covered a total period of 10 weeks and was carried out under the Mexican Standard, and the “Guide for the Care and Use of Laboratory Animals” of the National Research Council and Health Institutes was followed [80,81].

At first, animals were housed in polypropylene cages under conditions of controlled temperature (20–25 °C), relative humidity (36–60%), and light/dark cycles, with water ad libitum. Before starting the experiment, they were given a one-week acclimatization period to adapt to their new habitat. Once finalized, animals were fed an HFD for 3 weeks to generate insulin resistance.

The HFD was made using butter, standard food (LabDiet 5001), and water at a ratio of 10:24:5 to obtain feed with a total caloric value of approximately 4900 Kcal/Kg from fats (58%), carbohydrates (27.5%), and proteins (14.5%) [82,83].

After this period, the animals fasted for 12 h and then were administered intraperitoneally with a single dose of 35 mg/Kg of STZ dissolved in a buffer of 0.1 M citrate, pH 4.5 (prepared on the same day of administration). Once administered, the hypercaloric diet was maintained, and 10% sucrose solution was placed on demand for 2 days.

One week after administration, postprandial blood glucose levels were measured by venipuncture using a glucometer. Those animals that maintained a glucose level between 200 and 400 mg/dL were included in the study [82,83]. Animals that were successfully induced to develop T2D were randomly separated into 4 distinct groups to be administered the corresponding treatments (according to Table 10) for 5 weeks [57].

##### Acute Evaluation

Once the experimental groups were formed, a fixed schedule was established for the daily oral administration of the respective treatments. These were prepared in a 2% Tween 80 suspension in water and administered via esophageal cannula. On the third day of treatment, postprandial blood glucose was measured with a glucometer Freestyle^®^ (Abbott Diabetes Care, Alameda, CA, USA), extracting a drop of blood from the caudal vein of each rat. The corresponding treatment was then administered, and glucose measurements were continued at 30, 60, 90, and 120 min, following the same procedure [43,83,84].

##### Subchronic Evaluation

**Figure 14:**
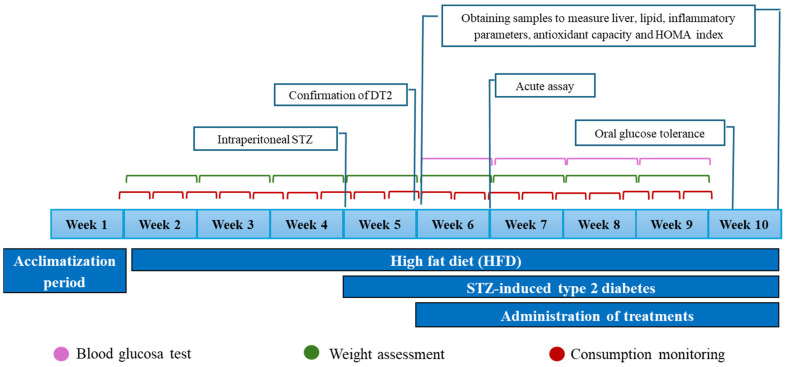
In vivo model scheme.

Treatments were administered daily as described above, adjusting the dose according to the weight of the individuals. Fasting glucose levels (6 h fast) were monitored every 7 days until the end of treatments at week 10 (Figure 14). Halfway through the last week of treatment, the OGTT was performed, administering a dose of 1.5 g/Kg of glucose orally and subsequently monitoring capillary blood glucose at 0, 15, 30, 60, 90, and 120 min [62]. In addition, a 24-element blood chemistry was measured in a Celercare V automated dry chemistry analyzer (Tianjin MNCHIP Technologies Co., Ltd., Tianjin, China) at the beginning and end of the model [83,84,85]. For this purpose, the blood samples were obtained from the tail of the rat.

### 3.5. Ex Vivo

At the end of the study, animals fasted for 12 h and were taken to a humane endpoint, where blood samples were obtained. These were kept at room temperature for 30 min and then centrifuged at 4000 RPM for 15 min at 4 °C. Finally, the serum was used to perform 24-element blood chemistry and quantify Trolox-equivalent TAC [86], TNF-α [87], and IL-6 levels [88] using commercial kits from Merck (Darmstadt, Germany), Sigma-Aldrich (St. Louis, MO, USA), Cat. No. MAK334-1KT, RAB0479-1KT, and RAB0311-1KT, respectively. In addition, insulin was quantified using the Rat Ins1/Insulin ELISA Kit, from Sigma-Aldrich, No. Cat. RAB0904-1KT [89] to determine the HOMA-IR index [15]. All kits were performed according to the manufacturer’s instructions without modifications.

### 3.6. Statistical Analysis

Data obtained in vivo studies are expressed as mean ± standard error of the mean (SEM) with *n* = 6. For all parameters, comparisons between groups were carried out using a two-way analysis of variance (ANOVA) followed by Tukey’s multiple comparisons test. All statistical tests were performed and graphed using GraphPad 8.2.1 software, where *p* < 0.05 was considered to indicate whether there was a significant difference in each group concerning time and between each treatment.

## 4. Conclusions

In summary, the compounds benzothiazolisothioureas evaluated in silico have optimal physicochemical, pharmacokinetic, and toxicological properties for oral administration, complying with medicinal chemistry filters and showing a significant affinity for ALR2 and PPAR-γ by hydrogen bonds of carbonyl groups that stabilize the interaction with both molecular targets. From the in silico analysis, compounds were selected and successfully synthesized and characterized. In vitro assays confirmed the inhibition capacity of ALR2 for compounds **8c** and **8d**, comparable to or superior to the reference drug, although no antioxidant effect was evidenced. In vivo studies showed the safety of the synthesized compound with an LD_50_ > 1250 mg/kg. Finally, compound **8d** exhibited a sustained antihyperglycemic effect over 5 weeks of treatment in Wistar rats with an HFD and STZ-induced T2D. This effect is associated with PPAR-γ agonism, as it improved insulin resistance and reduced dyslipidemia and polydipsia, without causing hepatotoxicity or other relevant adverse effects, although **8d** did not show intrinsic antioxidant or anti-inflammatory activity in the evaluated model. Together, these findings position compound **8d** as a promising candidate for the development of adjuvant agents in the treatment of T2D. Nevertheless, further preclinical trials are needed to clearly demonstrate the antidiabetic effect and elucidate its underlying mechanisms of action.

## Figures and Tables

**Table 3:** In silico toxicity prediction.

	Toxicity prediction					
	Mutagenicity	Cytotoxicity	Reproductive Effect	Irritant Effect	LD_50_	Class
**5d**	×	×	×	×	1000	IV
**8c**	×	×	×	×	1000	IV
**8d**	×	×	×	×	1000	IV
**12d**	×	×	×	×	1000	IV
**Epa**	×	×	×	×	1365	IV
**Zop**	×	×	×	×	5	II
**PGZ**	×	×	×	×	1000	IV

Data obtained from PorTox 3.0 (https://tox.charite.de/protox3/, accessed on 06 January 2024) [39]. LD_50_, median lethal dose.

**Table 5:** Weights of organs obtained from necropsy after the acute oral toxicity procedure from top to bottom.

	Vehicle*	8c	8d	12d
**IWA**	238.00	306.20 ± 9.70	201.0 ± 7.17	208.47 ± 6.47
**FWA**	331.50	204.37 ± 1.47	310.57 ± 11.6	309.67 ± 4.67
**Spleen**	0.80	0.73 ± 0.03	0.67 ± 0.07	0.83 ± 0.03
**Liver**	19.00	18.87 ± 1.47	17.57 ± 0.50	19.33 ± 0.03
**Intestine**	24.70	30.20 ± 1.50	29.97 ± 0.72	29.10 ± 0.50
**Stomach**	2.00	1.57 ± 0.07	1.70 ± 0.10	1.53 ± 0.07
**Kidney**	3.20	2.57 ± 0.27	2.97 ± 0.03	2.87 ± 0.067
**Heart**	1.60	1.07 ± 0.17	1.30 ± 0.03	1.23 ±0.13

IWA, initial weight of animals; FWA, final weight of animals. Vehicle*: 2% Tween 80 in water; compounds **8c**, **8d**, and **12d** were administered at 1250 mg/Kg. Data represented as the mean ± SEM.

**Table 6:** Insulin resistance and sensitivity index of the groups at the beginning and end of treatment.

		Healthy	Diabetic	T2D + PGZ	T2D + 8d
HOMA-IR	Initial	1.97 ± 0.44	2.68 ± 0.57	2.59 ± 0.34	4.22 ± 1.58
	Final	1.41± 0.40	5.42 ± 1.85	2.32 ± 0.41	1.66 ± 0.61

Values represented as the mean ± SEM with *n* = 4. All of them are without statistical difference (*p* > 0.05).

**Table 7:** Total antioxidant capacity equivalent to Trolox.

Group	TAC Equivalent to Trolox [μM]
Healthy control	113.29 ± 8.39
Diabetic control	87.49 ± 22.28
T2D + PGZ	97.75 ± 1.29
T2D + **8d**	79.26 ± 17.28

Values represented as the mean ± SEM with *n* = 4. All of them are without statistical difference (*p* > 0.05).

**Table 8:** Concentration of IL-6 in serum from the groups.

Group	IL-6 [pg/mL]
Healthy control	373.75 ± 130.19
Diabetic control	536.82 ± 136.49
T2D + PGZ	234.25 ± 52.05
T2D + **8d**	887.53 ± 243.91

Values represented as the mean ± SEM with *n* = 4. All of them are without statistical difference (*p* > 0.05).

**Table 9:** TNF-α concentration in serum from the groups.

Group	TNF-α [pg/mL]
Healthy control	1641.41 ± 266.06
Diabetic control	4452.61 ± 294.68
T2D + PGZ	3000.53 ± 519.24
T2D + **8d**	5417.72 ± 2104.2

Values represented as the mean ± SEM with *n* = 4. All of them are without statistical difference (*p* > 0.05).

**Table 10:** Distribution of groups and characteristics of their food and treatment administration.

Group	Food	Treatment
Healthy	LabDiet 5001	None
Diabetic	HFD	None
T2D + PGZ	HFD	PGZ (30 mg/Kg) *
T2D + **8d**	HFD	Compound **8d** (32 mg/Kg)

T2D, type 2 diabetes; HFD, high-fat diet; * PGZ, 30 mg Aurax^®^, Farmacia San Pablo, CDMX, México.

## Data Availability

All the relevant data found in the study are available in the article. The data supporting the study are in the Supplementary Materials section.

## Supplementary Materials

The following supporting information can be downloaded at https://www.mdpi.com/article/10.3390/molecules30163427/s1, Figure S1: ^1^H NRM (400 MHz, CDCl_3_) of compound **2**; Figure S2: ^13^C NRM (101 MHz, CDCl_3_) of compound **2**; Figure S3: FTIR-ATR of compound **2**; Figure S4: SM (DART+) of compound **2**; Figure S5: HPLC analysis of compound **2**; Figure S6: SM (ESI+) of compound **5d**; Figure S7: HPLC analysis of compound **5d**; Figure S8: ^1^H NRM (400 MHz, CDCl_3_) of compound **8d**; Figure S9: ^13^C NRM (101 MHz, CDCl_3_) of compound **8d**; Figure S10: FTIR-ATR of compound **8d**; Figure S11: SM (DART+) of compound **8d**; Figure S12: SM (ESI+) of compound **8d**; Figure S13: HPLC analysis of compound **8d**; Figure S14: ^1^H NRM (400 MHz, CDCl_3_) of compound **12d**; Figure S15: ^13^C NRM (101 MHz, CDCl_3_) of compound **12d**; Figure S16: FTIR-ATR of compound **12d**; Figure S17: SM (DART+) of compound **12d**; Figure S18: SM (ESI+) of compound **12d**; Figure S19: HPLC analysis of compound **12d**; Figure S20: ^1^H NRM (400 MHz, CDCl_3_) of compound **8c**; Figure S21: ^13^C NRM (101 MHz, CDCl_3_) of compound **8c**; Figure S22: FTIR-ATR of compound **8c**; Figure S23: SM (DART+) of compound **8c**; Figure S24: SM (ESI+) of compound **8c**; Figure S25: HPLC analysis of compound **8c**; Figure S26: Comparison of the inhibition percentages; Figure S27: Oral glucose tolerance test (OGTT). Effect of treatments on blood glucose levels after a single oral administration of 1.5 g/Kg of glucose. Data reported as the mean ± SEM with *n* = 4. * Significant difference against healthy control (*p* < 0.05); ns, no significant difference against healthy control (*p* > 0.05). Table S1: Principal physicochemical properties; Table S2: Drug-likeness and medicinal chemistry filters to determine the viability of molecules to be developed as an oral drug; Table S3: Pharmacokinetic properties; Table S4: Chronic toxicity prediction of compounds; Table S5: Values of Gibbs free energy (ΔG), inhibition constant (Ki), and essential amino acid residues of the active site with which the compounds interact; Table S6: Relative inhibition of the compounds tested on the ALR2 enzyme, measured from the rate of reduction in the NADPH cofactor with the kit (ab273276); Table S7: Percentage reduction in the ferric ion to the ferrous ion in the presence of the different compounds. (FRAP assay); Table S8: Percentage reduction in radical cation ABTS in the presence of the different compounds (ABTS assay); Table S9: Values of the parameters measured in the 24-element blood chemistry; Table S10: Parameters obtained after the induction of T2D before treatment; Table S11: Parameters obtained at the end of the evaluation period after 5 weeks of treatment.

## Abbreviations

The following abbreviations are used in this manuscript:

ALR2	Aldose reductase
PPAR-γ	Peroxisome proliferator-activated receptor gamma
HFD	High-fat diet
STZ	Streptozotocin
LD_50_	Median lethal dose
DM	Diabetes mellitus
IDF	International Diabetes Federation
PGZ	Pioglitazone
T2D	Type 2 diabetes
BBB	Blood–brain barrier
MW	Molecular weight
cLogP	Octanol/water partition coefficient calculated
nON	Number of hydrogen bond donors
nOHNH	Number of hydrogen bond acceptors
Nrot	Number of rotal link donors
TPSA	Topological polar surface area
LogS	Logarithm of the aqueous solubility
MR	Molar refractivity
Zop	Zopolrestat
Epa	Epalrestat
Caco-2	Permeability through cells derived from human colon adenocarcinoma
MDCK	Through Madin–Darby canine kidney cells
NADP+/NADPH	Oxidized/reduced nicotinamide adenine dinucleotide phosphate
P-gp	P-glycoprotein
OATP1B1	Organic anion transporting polypeptide 1B1
BSEP	Bile salt export pump
BCRP	Breast cancer resistance
CYP450	Cytochrome P-450
%ABS	Oral absorption percentage
HIA	Human intestinal absorption
GHS	Globally Harmonized System
ABP	Anion-binding pockets
ΔG	Gibbs free energy
Ki	Inhibition constant
#IT	Number of total interactions
# I-ABP	Number of interactions with anion-binding pocket residues
LBD	Ligand-binding domain
TBAH	Tetrabutylammonium hydroxide
FRAP	Ferric reducing antioxidant power
AOT	Acute oral toxicity
OECD	Organization for Economic Co-operation and Development
SEM	Standard error of the mean
HbA1c	Glycated hemoglobin
HOMA-IR	Homeostatic model assessment for insulin resistance
T-chol	Total cholesterol
TG	Triglyceride
ALT	Alanine aminotransferase
AST	Aspartate aminotransferase
GGT	Gamma-glutamyltransferase
ALP	Alkaline phosphatase
BUN	Blood urea nitrogen
CK	Creatine kinase
TAC	Total antioxidant capacity
IL-6	Interleukin-6
TNF-α	Tumor necrosis factor alpha
R^2^	Coefficient of determination
MMFF94	Merck molecular force field
TLC	Thin-layer chromatography
5-ASA	5-aminosalicylic acid
ANOVA	A two-way analysis of variance
PAINS	Pan-assay interference compounds
AUC	Area under the curve
TP	Total protein
ALB	Albumin
GLO	Globulin
TBIL	Total bilirubin
TBA	Total bile acids
AMY	Amylase
CRE	Creatinine
OGTT	Oral glucose tolerance test
Mp	Melting point
tr	Retention time
