# Supplementary material for: Preclinical Evaluation of 2-Aminobenzothiazole Derivatives: In Silico, In Vitro, and Preliminary In Vivo Studies as Diabetic Treatments and Their Complications
Source: Molecules. 2025 Aug 20;30(16):3427. doi: 10.3390/molecules30163427 (PMC12388506; doi:10.3390/molecules30163427)
Supplement: Supplementary file 1 [file molecules-30-03427-s001.zip › molecules-3836048-supplementary.pdf]

molecules

MDPI

Supplementary Material

Preclinical Evaluation of 2-Aminobenzothiazole Derivatives: In Silico, In Vitro, and Preliminary In Vivo Studies as Diabetic Treatments and Their Complications

Natalia Reyes-Vallejo [1], Miguel Valdes [1,2,3,*], Adelfo Reyes-Ramírez [4], Juan Andres Alvarado-Salazar [1], Alejandro Cruz [5], Erik Andrade-Jorge [6] and Jessica Elena Mendieta-Wejebe [1,*]

[1] Laboratorio de Biofísica y Biocatálisis, Sección de Estudios de Posgrado e Investigación, Escuela Superior de Medicina, Instituto Politcnico Nacional. Plan de San Luis y Salvador Díaz Mirón s/n, Casco de Santo Tomás, Miguel Hidalgo, Mexico City 11340, Mexico; reyes.natalia4@gmail.com (N.R.-V.); andres.alvarado.salazar@comunidad.unam.mx (J.A.A.-S.)
[2] Unidad de Investigación Médica en Farmacología, UMAE Hospital de Especialidades 2 Piso CORSE Centro Médico Nacional Siglo XXI, Instituto Mexicano del Seguro Social, Av. Cuauhtémic 330, Col. Doctores, Mexico City 06720, Mexico
[3] Laboratorio de Inmunología, Departamento de Sistemas Biológicos, Universidad Autónoma Metropolitana Unidad Xochimilco, Calz. del Hueso 1100, Coapa, Villa Quietud, Coyoacán, Mexico City 04960, Mexico
[4] Laboratorio de Síntesis Farmacéutica, Unidad Multidisciplinaria de Investigación Experimental Zaragoza, FES Zaragoza-Universidad Nacional Autonoma de México, Campus II. Batalla 5 de Mayo s/n, Ejército de Oriente Zona Peñón, Iztapalapa, Mexico City 09230, Mexico; adelfo.reyes@zaragoza.unam.mx
[5] Laboratorio de Química Supramolecular y Nanociencias, Unidad Profesional Interdisciplinaria de Biotecnología, Departamento de Ciencias Básicas, Instituto Politécnico Nacional, Av. Acueducto s/n, Colonia Barrio La Laguna Ticomán, Mexico City 07340, Mexico; alcralmx@hotmail.com
[6] Laboratorio de Investigación en Bioquímica, Sección de Estudios de Posgrado e Investigación, Escuela Superior de Medicina, Instituto Politécnico Nacional. Plan de San Luis y Salvador Díaz Mirón s/n, Casco de Santo Tomás, Miguel Hidalgo, Mexico City 11340, Mexico; andrade136@hotmail.com
* Correspondence: valdesguevaramiguel@gmail.com (M.V.); jmendieta@ipn.mx (J.E.M.-W.); Tel.: +55-31184059 (M.V.); Tel.: +52-5557296000 (J.E.M.-W.)

Contents

1. Properties predicted by online platforms 2
2. Docking molecular data 7
3. Spectral characterization of products 9
4. In vitro results 23
5. In vivo results 24
6. Ex vivo results 26

1. Properties predicted by online platforms

Table S1. Principal physicochemical properties.

	Physicochemical properties										
	Lipinski rules				Aqueous solubility						
	MW	LogP	nON	nOHNH	nrot	TPSA	logS	[mg/mL]	Class	MR	Volume [Å³]
**5a**	281.36	1.12	5	2	5	74.6	-3.33	$1.31E^{-01}$	II	75.41	253.47
**5b**	295.39	0.91	5	2	5	74.6	-3.65	$6.59E^{-02}$	II	80.22	270.77
**5c**	357.46	3.62	5	2	6	74.6	-4.89	$4.56E^{-02}$	III	99.9	340.78
**5d**	371.49	2.37	5	2	7	74.6	-5.09	$3.05E^{-03}$	III	104.7	358.08
**8a**	295.39	2.65	5	1	6	63.6	-3.53	$8.62E^{-02}$	II	79.73	270.77
**8b**	309.42	2.98	5	1	6	63.6	-3.86	$4.25E^{-02}$	II	84.54	288.06
**8c**	371.49	4.23	5	1	7	63.6	-5.11	$2.92E^{-03}$	III	104.2	358.08
**8d**	385.51	4.44	5	1	8	63.6	-5.29	$1.97E^{-03}$	III	109.0	375.37
**11a**	264.31	1.24	6	3	4	86.6	-2.52	$7.97E^{-01}$	II	70.63	264.07
**11b**	278.34	1.07	6	3	4	86.6	-2.84	$4.02E^{-01}$	II	75.43	263.25
**11c**	340.41	2.53	6	3	6	86.6	-4.09	$2.77E^{-02}$	III	95.11	333.27
**11d**	354.44	2.32	6	3	5	86.6	-4.27	$1.88E^{-02}$	III	99.92	350.56
**12c**	370.50	3.48	5	2	6	66.4	-4.73	$6.84E^{-03}$	III	105.9	360.28
**12d**	384.53	3.69	5	2	7	66.4	-4.93	$4.55E^{-03}$	III	110.8	377.58
**ZOP**	419.38	3.54	6	1	5	85.1	-4.83	$6.27E^{-03}$	III	101.5	372.66
**EPA**	319.41	2.44	4	1	4	59.30	-4.19	$2.08E^{-02}$	II	91.76	304.18
**PGZ**	356.44	3.09	7	4	1	93.59	-4.31	$1.76E^{-02}$	III	102.6	357.29

Data obtained from Molinspiration: (https://www.molinspiration.com, accessed on 02 January 2024) and ADMETLab 3.0 (https://admetlab3.scbdd.com/server/evaluationCal, accessed on 02 January 2024). MW: Molecular Weight; cLogP: octanol/water partition coefficient calculated; nON: number of hydrogen bond donors; nOHNH: number of hydrogen bond acceptors; nrot: number of rotal link donors; TPSA: topological polar surface area; logS: logarithm of the aqueous solubility, with their respective classification as Class I; poorly soluble; Class II: moderately soluble; Class III: soluble; Class IV: very soluble; Class V: highly soluble; MR: Molar refractivity; Zop: zopolrestat; Epa: epalrestat; PGZ: pioglitazone

Table S2. Medicinal chemistry and drug-likeness filters to assess the oral viability of molecules

	Filters						
	Druglikeness				Medicinal chemistry		
	Ghose	Veber	Egan	Muegge	PAINS	Brenk	Leadlikeness
**5a**	√	√	√	√	√	2	√
**5b**	√	√	√	√	√	2	√
**5c**	√	√	√	√	√	2	2
**5d**	√	√	√	√	√	2	2
**8a**	√	√	√	√	√	2	√
**8b**	√	√	√	√	√	2	√
**8c**	√	√	√	√	√	2	2
**8d**	√	√	√	√	√	2	3
**11a**	√	√	√	√	√	2	√
**11b**	√	√	√	√	√	2	√
**11c**	√	√	√	√	√	2	√
**11d**	√	√	√	√	√	2	2
**12c**	√	√	√	√	√	2	2
**12d**	√	√	√	√	√	2	2
**Zop**	√	√	√	√	√	√	2
**Epa**	√	√	√	√	1	2	1
**PGZ**	√	√	√	√	√	1	2

Data obtained from ADMETLab 3.0 (https://admetlab3.scbdd.com/server/evaluationCal, accessed on 02 January 2024) and SwissADMET (https://www.swissadme.ch/, accessed on 03 January 2024). Ghose filter, Veber filter, Egan filter (Pharmacia), Muegge filter (Bayer): √: it meets all parameters; numbers are the amount of violation to each filter; PAINS: Pan-Assay Interference Compounds (structures that may present a false positive in activity); Brenk: structural alert (depict fragments of compounds that could be putatively toxic, chemically reactive, and metabolically unstable.

Table S3. Pharmacokinetic properties

	Absorption						Distribution				
	**%ABS**	**HIA**	**Caco-2**	**MDCK**	**PAMPA**	**P-gp**	**BBB**	**PPB (%)**	**VDss**	**OATP1B1 inhibitor**	**OATP1B3 inhibitor**
**5a**	83.27	93.01	-5.283	0	+	X	X	98.3	0.324	++	+++
**5b**	83.27	93.90	-5.139	0	++	X	X	98.3	0.366	+++	+++
**5c**	83.27	98.24	-5.132	0	+++	X	√	98.8	0.343	+++	+++
**5d**	83.27	98.24	-5.451	0	++	X	√	98.7	0.297	+++	+++
**8a**	87.06	96.79	-4.857	0	--	X	X	96.6	0.972	+++	+++
**8b**	87.06	97.20	-4.830	0	--	X	X	96.9	1.143	+++	+++
**8c**	87.06	98.17	-4.825	0	-	X	√	97.6	1.194	+++	+++
**8d**	87.06	98.06	-4.867	0	---	X	√	98.1	0.856	+++	+++
**11a**	79.12	87.65	-5.153	0	-	X	X	94.9	0.935	--	+++
**11b**	79.12	88.59	-5.353	0	+	X	X	94.6	1.257	+	+++
**11c**	79.12	92.41	-4.945	0	++	X	X	95.1	0.62	+	+++
**11d**	79.12	92.58	-5.270	0	--	X	X	95.7	0.83	++	+++
**12c**	86.10	95.80	-5.020	0	++	X	X	97.1	1.705	+++	+++
**12d**	86.10	95.75	-5.320	0	-	X	X	96.9	1.362	+++	+++
**Epa**	79.64	97.53	-5.369	0	--	X	X	90.4	0.34	---	--
**Zop**	88.54	99.52	-4.980	0	+++	X	X	98.1	0.453	--	++
**PGZ**	109.00	97.35	-4.907	0	-	X	X	99.0	0.688	+++	-

Data obtained from PREADMET (https://preadmet.webservice.bmdrc.org/, accessed on 02 January 2024), ADMETLab 3.0 (https://admetlab3.scbdd.com/server/evaluationCal, accessed on 02 January 2024) and SwissADMET (https://www.swissadme.ch/, accessed on 03 January 2024). %ABS: oral absorption percentage (¼ 109 – (0.345 TPSA)); HIA: human intestinal absorption (%); Caco2: permeability through cells derived from human colon adenocarcinoma (nm/s); MDCK: permeability through Madin-Darby canine kidney cells (nm/s); PAMPA: Parallel Artificial Membrane Permeability Assay; P-gp: P-glycoprotein substrate; BBB: blood–brain barrier penetration; PPB: Plasma Protein Binding; VDss: Volume distribution; OATP1B: Organic anion transporting polypeptide 1B1 and 1B3. X and -: it does not present, √ and +: it does present.

molecules

MDPI

Table S3 (continuation). Pharmacokinetic properties.

	Distribution			Metabolism						Excretion	
	BCRP inhibitor	MRP1 inhibitor	BSEP inhibitor	CYP450 inhibition CYP1A2	CYP2C19	CYP2C9	CYP2D6	CYP3A4	HLM Stability	Cl_{plasma} (ml/min/Kg)	T1/2 (h)
**5a**	---	+++	+++	X	X	√	X	X	+++	2.034	1.101
**5b**	---	+++	+++	X	X	√	X	X	+++	0.746	1.224
**5c**	---	-	+++	√	√	√	X	X	+	0.343	1.146
**5d**	---	+	+++	√	√	√	X	X	+++	1.482	1.032
**8a**	---	+	+++	√	√	√	X	X	+++	5.448	0.55
**8b**	---	+++	+++	√	√	√	X	X	+++	5.966	0.527
**8c**	---	+++	+++	√	√	√	X	X	+++	3.537	0.562
**8d**	--	+++	+++	√	√	√	X	X	+++	5.595	0.429
**11a**	---	+++	++	X	X	X	X	X	--	1.827	0.719
**11b**	---	+++	+++	√	X	X	X	X	--	1.875	0.861
**11c**	---	++	+++	√	√	√	X	X	-	1.229	0.922
**11d**	---	-	+++	√	√	√	X	X	+++	2.357	0.599
**12c**	---	--	++	X	X	X	X	X	++	4.01	0.485
**12d**	---	--	+++	X	X	X	X	X	++	6.182	0.388
**Epa**	---	+	+++	√	√	√	√	√	+++	2.019	1.142
**Zop**	---	+++	--	√	√	√	√	√	---	0.732	1.559
**PGZ**	---	--	+++	√	√	√	√	√	+++	7.71	0.753

BCRP: Breast Cancer Resistance Protein; MRP1: Multidrug Resistance-Associated Protein; BSEP: Bile Salt Export Pump; CYP450: cytochrome P-450; HLM: Human Liver Microsomes; Clplasma: Clearance; T1/2: Half-life drugs. X and -: it does not present, √ and +: it does present.

Table S4. Chronic toxicity prediction of compounds

	Toxicity prediction										
	M	T	RE	IE	LD_{50}	Class	Nephrotoxicity	Neurotoxicity	DILI	Respiratory	Hepatotoxicity
**5a**	X	X	X	X	1000	IV	0.3	0.182	0.854	0.345	0.727
**5b**	X	X	X	X	1000	IV	0.548	0.153	0.951	0.276	0.824
**5c**	X	X	X	X	1000	IV	0.533	0.191	0.956	0.28	0.818
**5d**	X	X	X	X	1000	IV	0.335	0.243	0.914	0.287	0.885
**8a**	X	X	X	X	1000	IV	0.355	0.444	0.724	0.264	0.767
**8b**	X	X	X	X	1000	IV	0.336	0.375	0.888	0.241	0.83
**8c**	X	X	X	X	1000	IV	0.411	0.564	0.925	0.253	0.844
**8d**	X	X	X	X	1000	IV	0.295	0.479	0.797	0.189	0.888
**11a**	X	X	X	X	1000	IV	0.415	0.404	0.807	0.15	0.769
**11b**	√	X	X	X	1000	IV	0.564	0.573	0.959	0.145	0.833
**11c**	X	X	X	X	1000	IV	0.61	0.706	0.907	0.166	0.858
**11d**	X	X	X	X	1000	IV	0.295	0.479	0.797	0.234	0.868
**12c**	X	X	X	X	999	IV	0.514	0.755	0.874	0.234	0.824
**12d**	X	X	X	X	1000	IV	0.564	0.444	0.959	0.145	0.833
**Epa**	X	X	X	X	1365	IV	0.398	0.27	1	0.707	0.574
**Zop**	X	X	X	X	5	II	0.961	0.731	0.999	0.525	0.844
PGZ	X	X	X	X	1000	IV	0.056	0.933	0.988	0.917	0.587

Data obtained from PorTox 3.0 (https://tox.charite.de/protox3/, accessed on 06 January 2024) and ADMETLab 3.0 (https://admetlab3.scbdd.com/server/evaluationCal, accessed on 02 January 2024). M: mutagenicity; T: tumorigenicity; IE: irritant effects; RE: reproductive effects √: it does present the effect; X: it does not present; LD_{50}: Lethal dose 50 (mg/kg); DILI: Drug Induced Liver Injury.

molecules

MDPI

2. Docking molecular data

Table S5. Values of Gibbs free energy (ΔG), Inhibition constant (Ki) and essential amino acid residues of the active site with which the compounds interact.

	ALR2 (4JIR)															
	**ΔG (Kcal/mol)**	**Ki (μM)**	**#IT**	**#I-ABP**	**Trp20**	**Val47**	**Tyr48**	**His110**	**Trp111**	**NADPH**	**Phe122**	**Trp219**	**Ala299**	**Leu300**	**Leu301**
**5a**	-7.05	2.6100	12	4					√	√	√	√	√	√	√
**5b**	-7.35	1.4900	11	2	√				√	√	√	√		√	√
**5c**	-8.84	0.2817	9	2					√	√	√	√	√	√	√
**5d**	-7.98	0.6026	11	1						√	√	√		√	√
**8a**	-7.63	1.5000	12	0							√	√	√	√	√
**8b**	-7.29	1.7700	8	0										√	√
**8c**	-8.14	0.3789	9	2					√	√	√	√	√	√	√
**8d**	-8.39	0.2618	13	1						√	√	√	√	√	√
**11a**	-7.23	1.8300	14	2		√			√	√	√	√	√	√	√
**11b**	-7.34	1.0500	16	1					√	√	√	√	√	√	√
**11c**	-8.31	0.2690	21	8	√	√		√	√	√	√	√	√	√	√
**11d**	-8.75	0.1621	17	5		√			√	√	√	√	√	√	√
**12c**	-9.16	0.1244	18	1					√	√	√	√	√	√	√
**12d**	-8.62	0.1714	16	3	√				√	√	√	√	√	√	√
**Epa**	-8.50	0.3592	23	7	√					√	√	√	√	√	√
**Zop**	-8.40	0.3280	19	9	√	√	√	√	√	√	√	√	√	√	√
**PGZ**	-9.38	0.0665	8	6		√			√		√	√			

#IT: Number of total interactions; # I-ABP: Number of interactions with anion-binding pocket residues; √: it does present the effect.

molecules

MDPI

Table S5 (continuation). Values of Gibbs free energy (ΔG), Inhibition constant (Ki) and essential amino acid residues of the active site with which the compounds interact

	PPAR-γ (2FVJ)							
	**ΔG (Kcal/mol)**	**Ki (μM)**	**#IT**	**#I-LBD**	**Cys285**	**Arg288**	**Ser289**	**His449**
**5a**	-5.89	43.6	10	4	√			
**5b**	-6.01	21.98	13	2		√		
**5c**	-7.78	1.23	14	5	√	√		
**5d**	-8.45	0.338	18	6	√	√		√
**8a**	-6.02	27.86	12	1		√		
**8b**	-6.48	15.23	11	4		√		
**8c**	-8.8	0.1223	19	0				
**8d**	-7.77	0.5502	16	3		√		
**11a**	-6.22	16.99	11	5	√	√		√
**11b**	-6.31	9.71	12	1		√		
**11c**	-7.88	0.81237	15	5	√			√
**11d**	-7.65	1.14	18	8				
**12c**	-8.98	0.14284	18	0				
**12d**	-7.97	0.5406	17	4				
**Epa**	-7.79	1.49	15	5	√			√
**Zop**	-8.08	0.5108	23	5	√		√	√
**PGZ**	-9.48	0.0994	11	4	√	√		

#IT: Number of total interactions; # I-ABP: Number of interactions with anion-binding pocket residues; √: it does present the effect

3. Spectral characterization of products

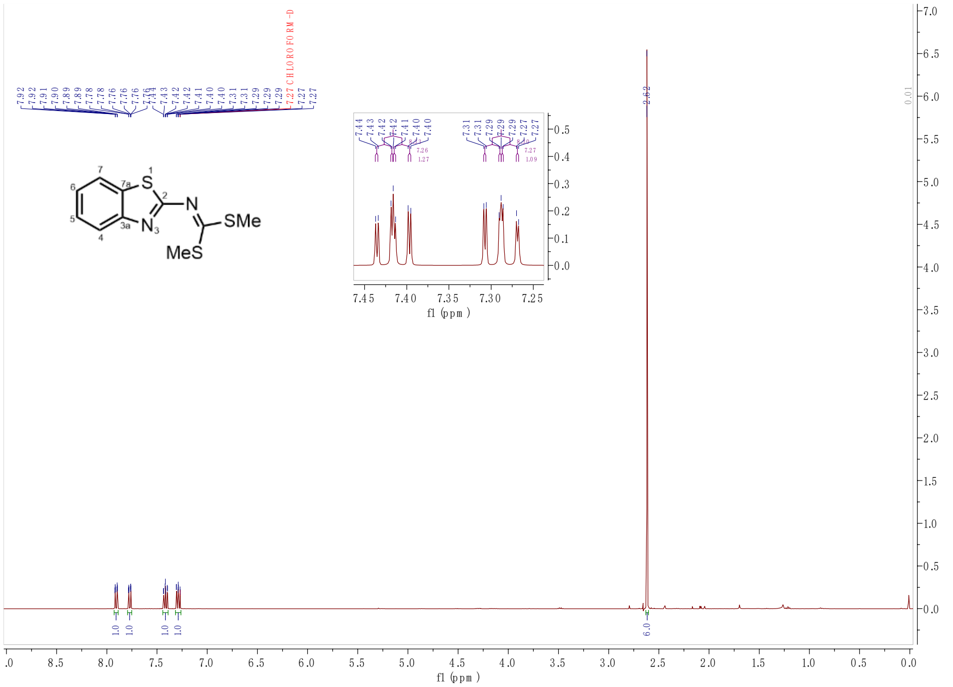

Figure S1. ^{1}H NRM (400 MHz, $CDCl_3$) of compound **2**.

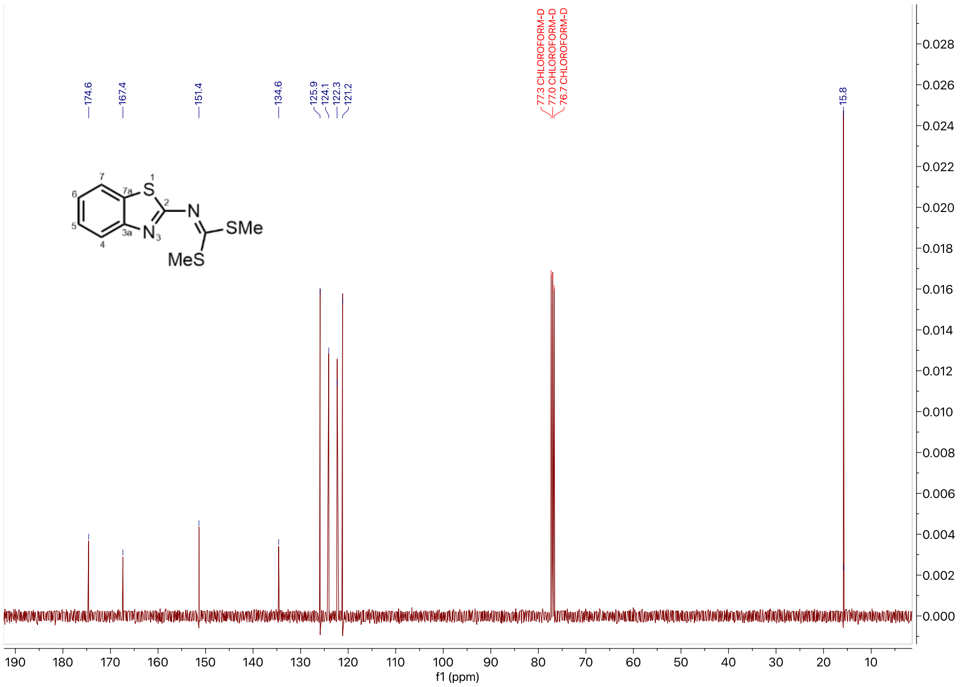

Figure S2. ^{13}C NRM (101 MHz, $CDCl_3$) of compound **2**.

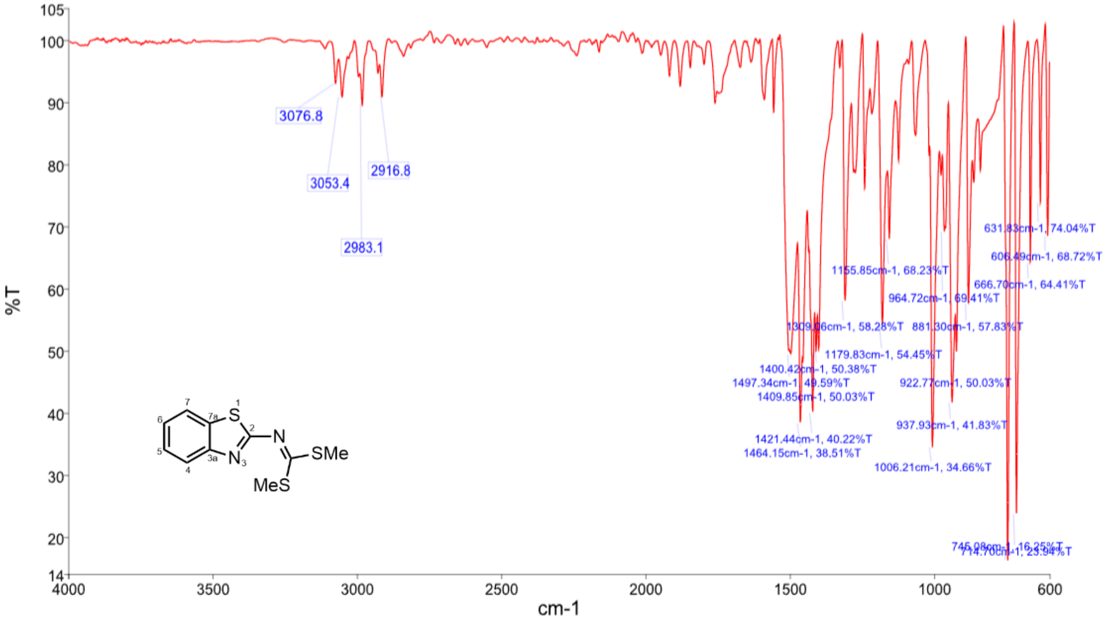

Figure S3. FTIR-ATR of compound **2**.

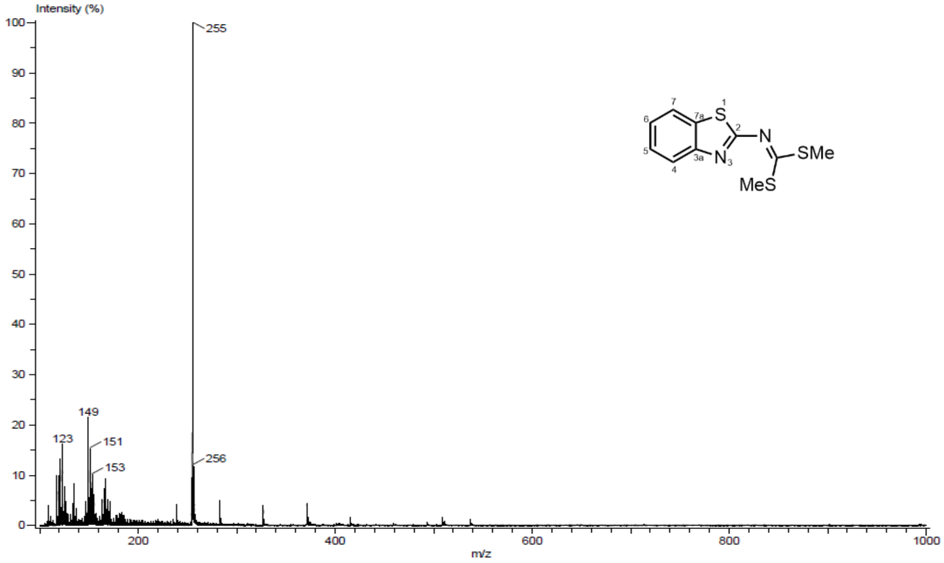

Figure S4. SM (DART+) of compound **2**.

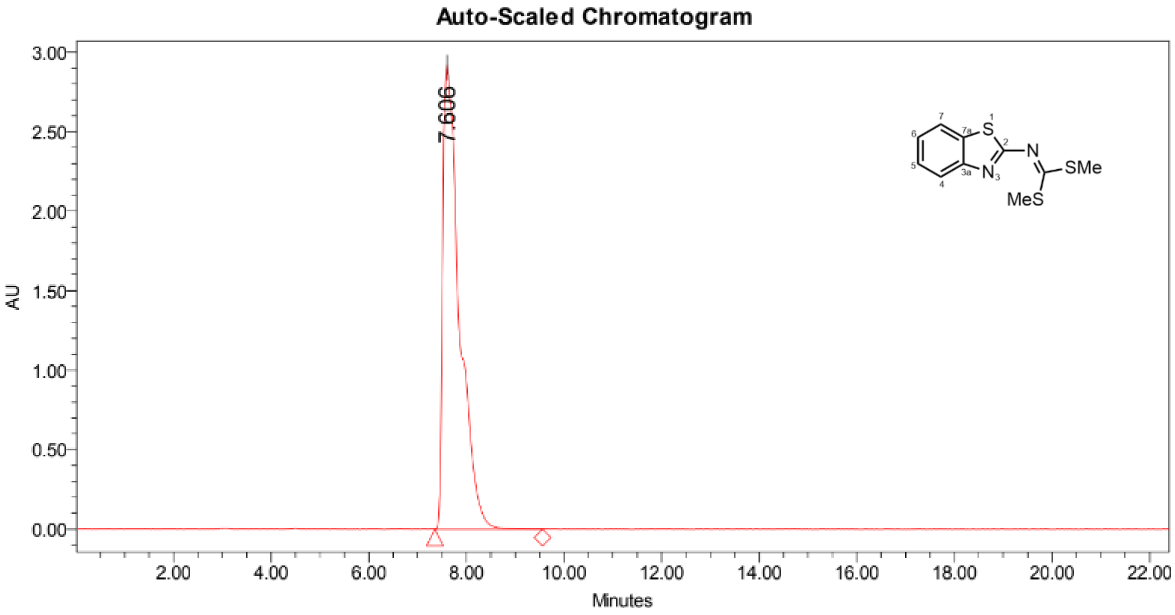

Figure S5. HPLC analysis of compound **2**.

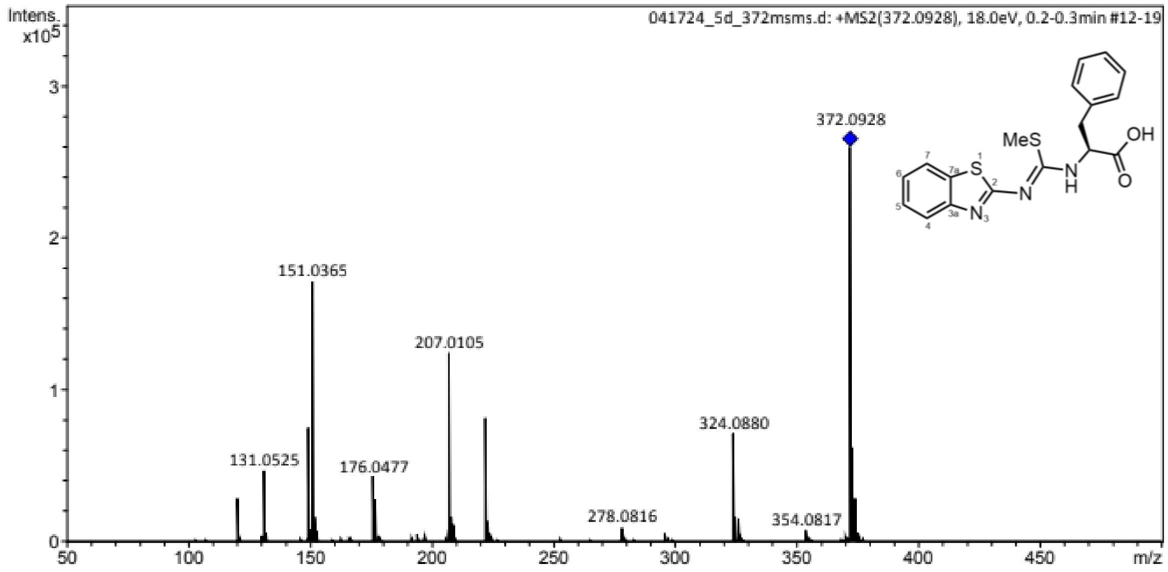

Figure S6. SM (ESI+) of compound **5d**.

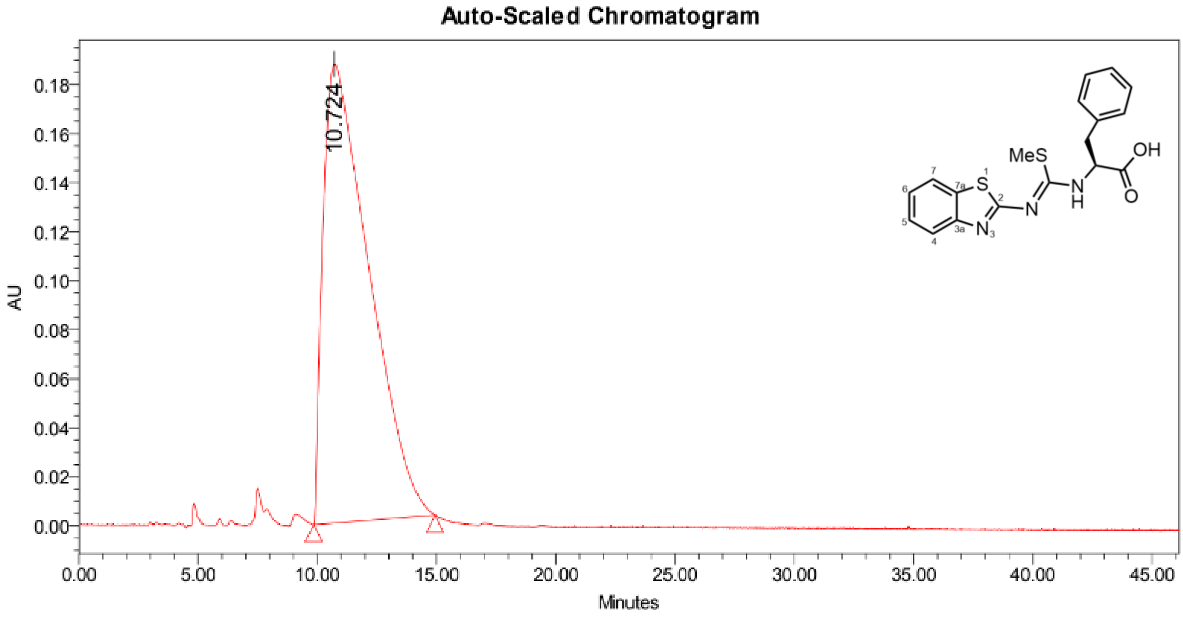

Figure S7. HPLC analysis of compound **5d**.

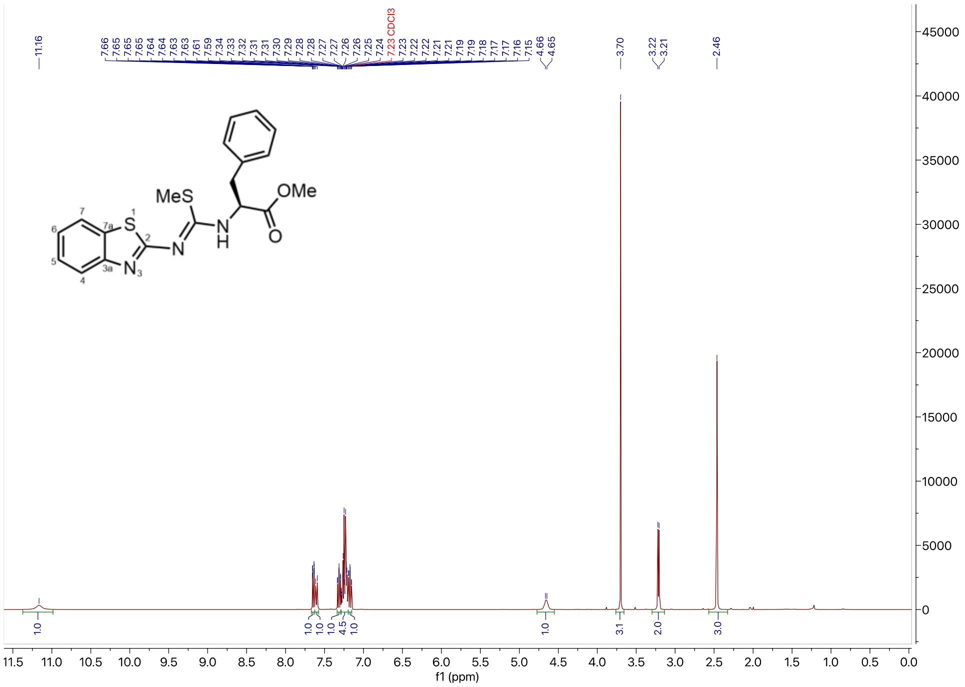

Figure S8. ^{1}H NRM (400 MHz, $CDCl_3$) of compound **8d**.

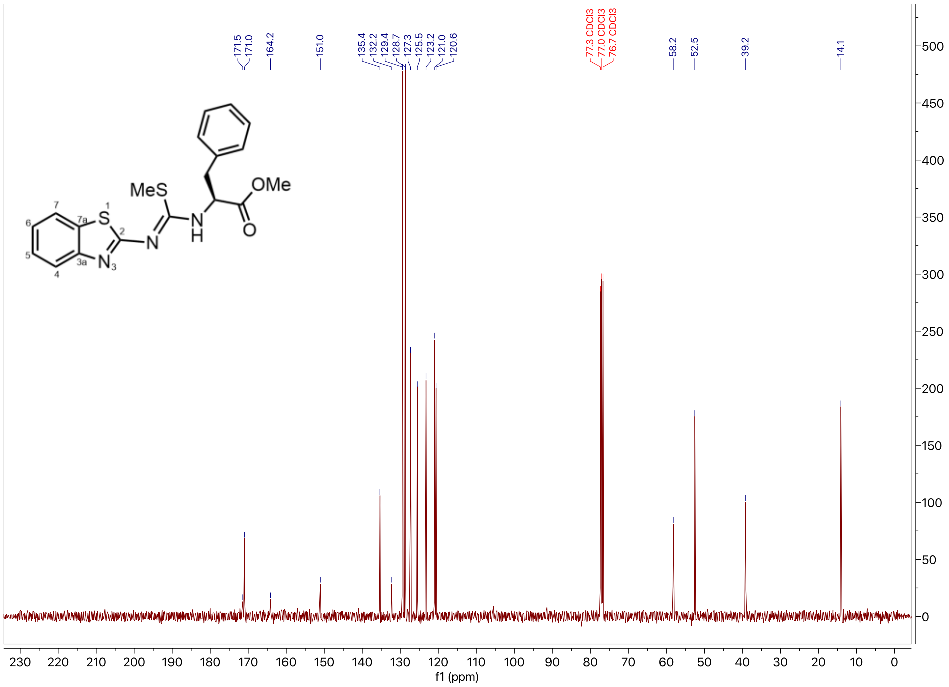

Figure S9. ^{13}C NRM (101 MHz, $CDCl_3$) of compound **8d.**

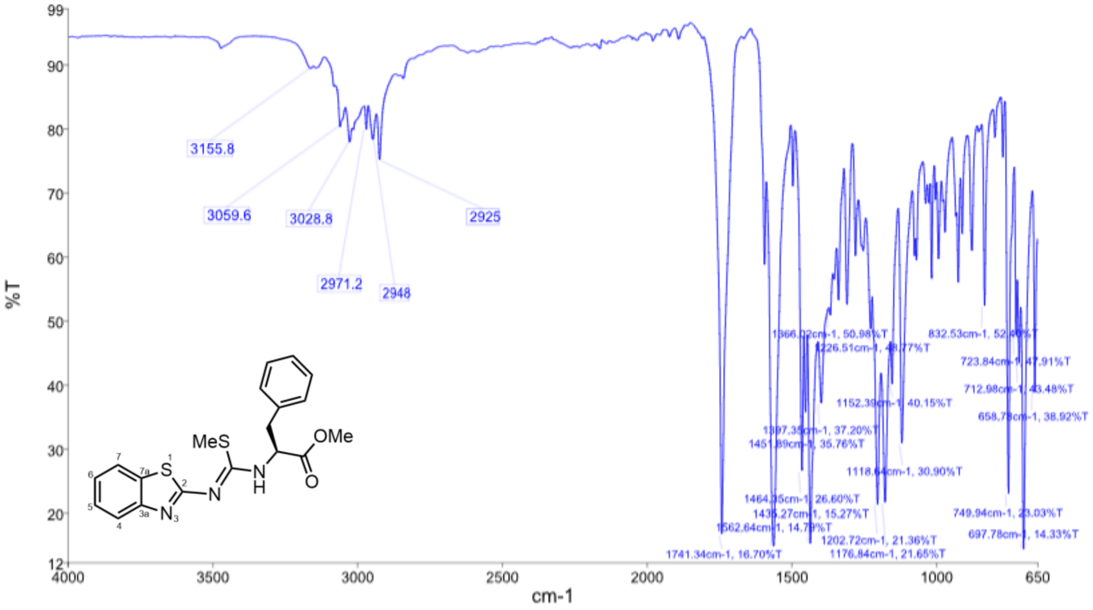

Figure S10. FTIR-ATR of compound **8d**.

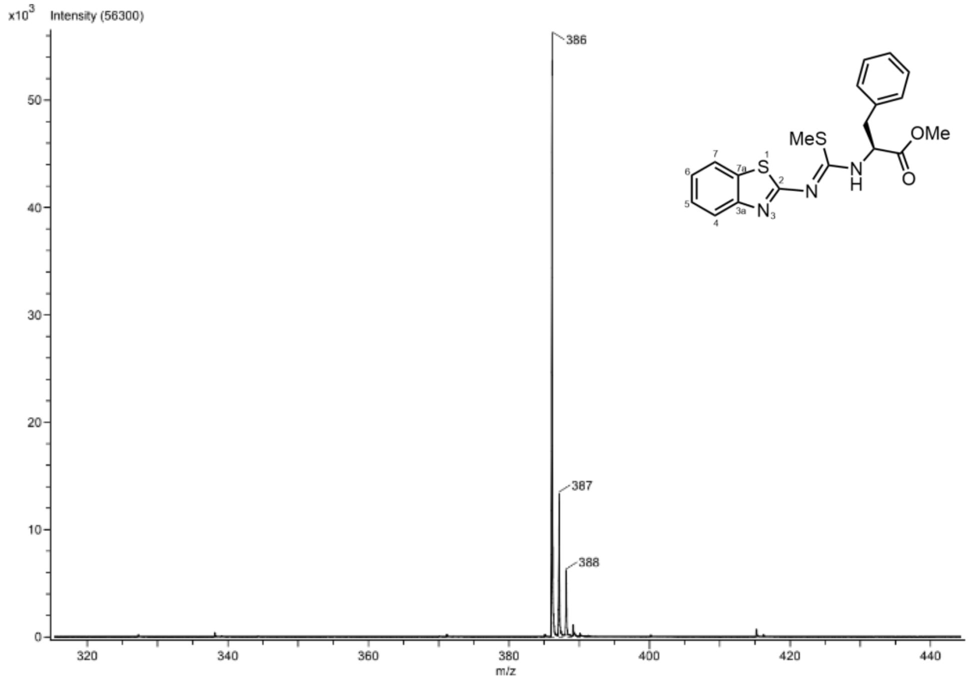

Figure S11. SM (DART+) of compound **8d**.

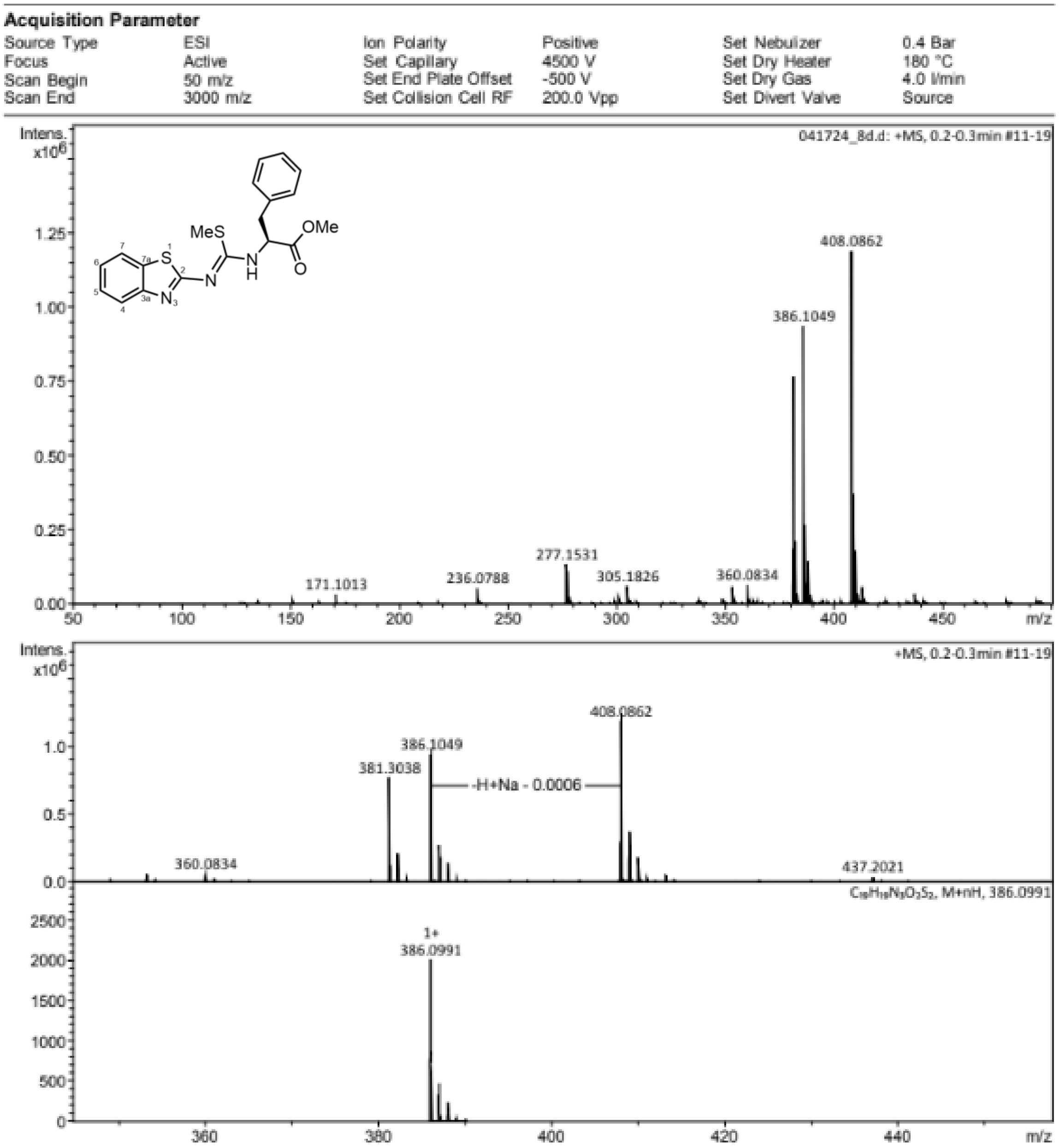

Figure S12. SM (ESI+) of compound **8d**.

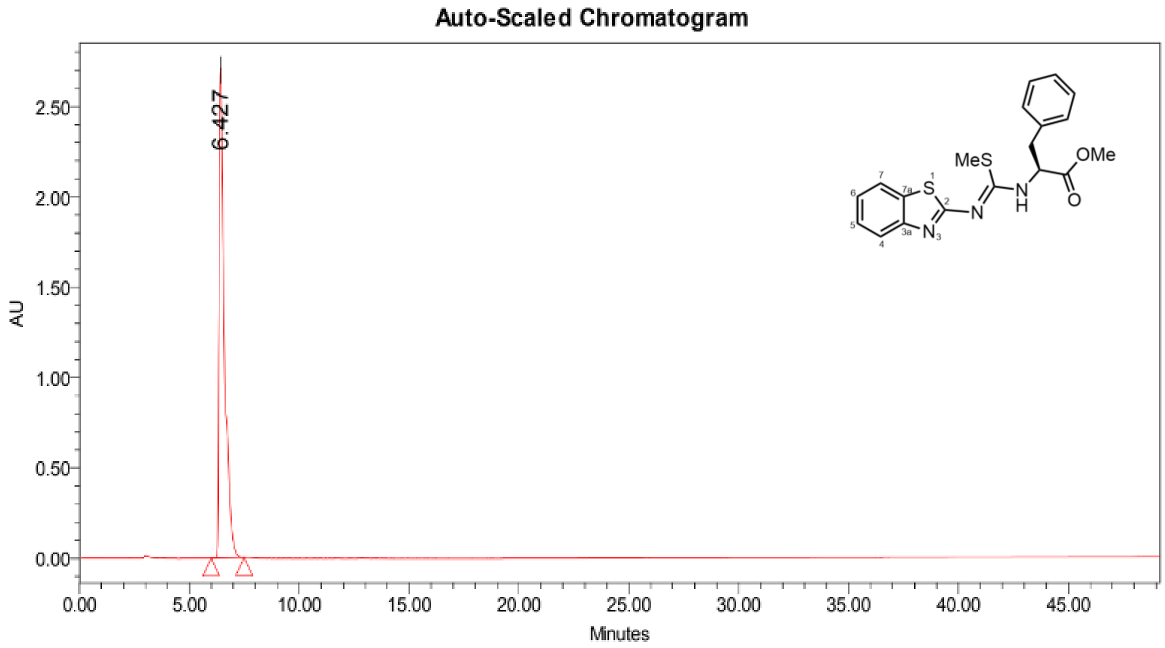

Figure S13. HPLC analysis of compound **8d**.

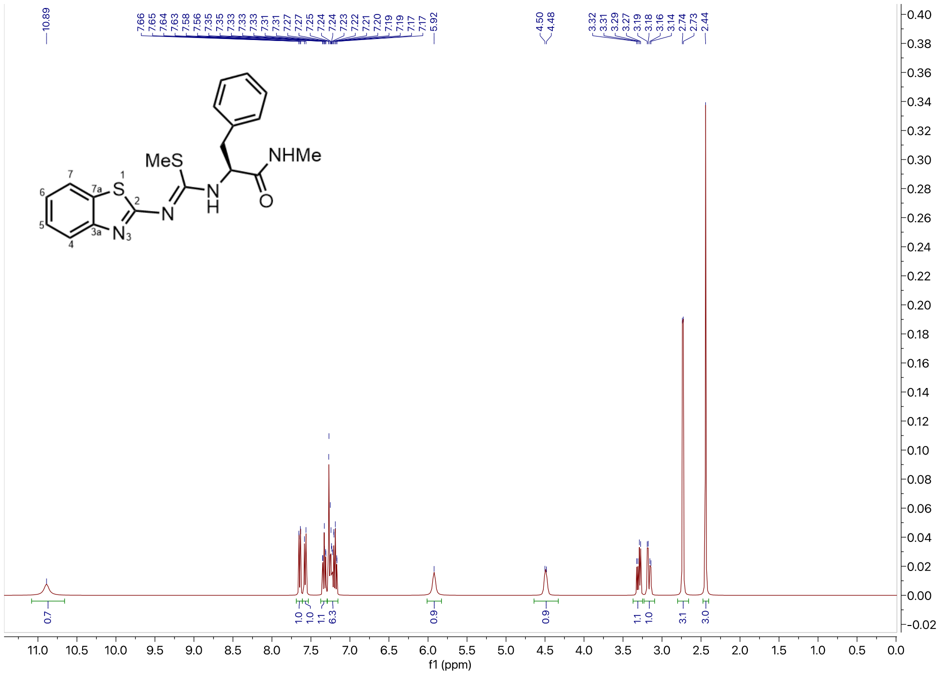

Figure S14. ^1H NRM (400 MHz, $CDCl_3$) of compound **12d**.

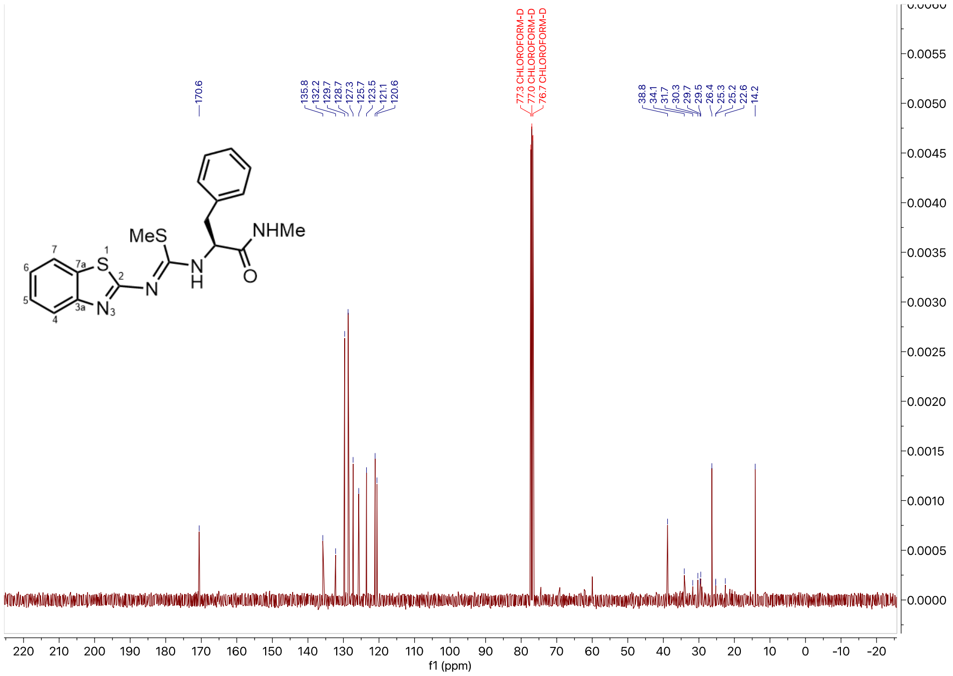

Figure S15. ^{13}C NRM (101 MHz, $CDCl_3$) of compound **12d**.

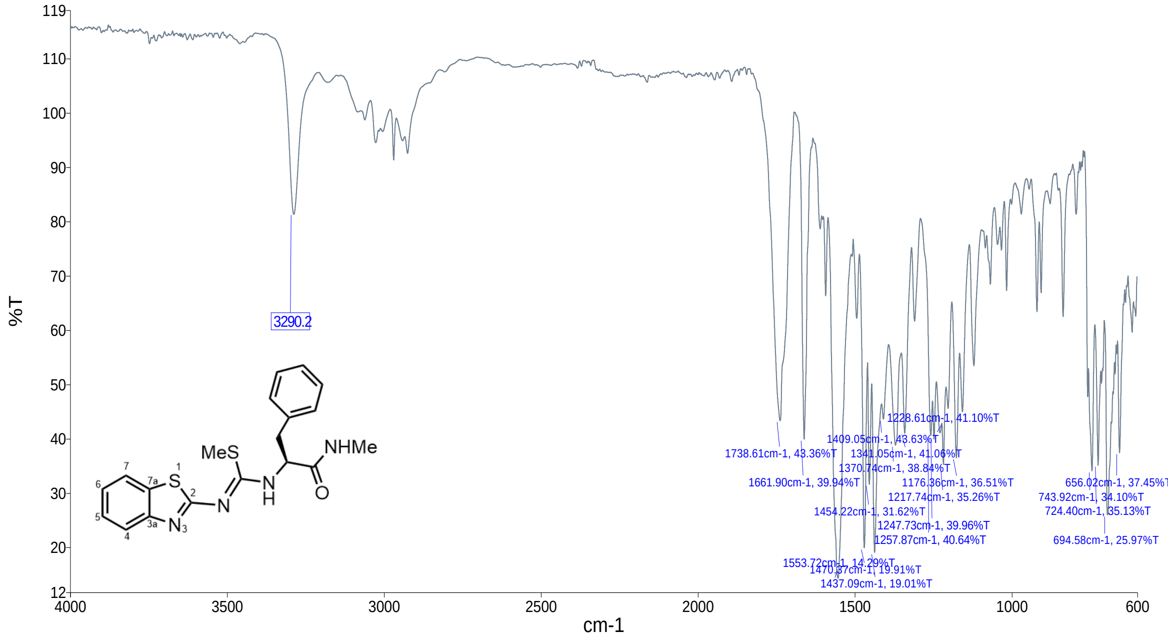

Figure S16. FTIR-ATR of compound **12d**.

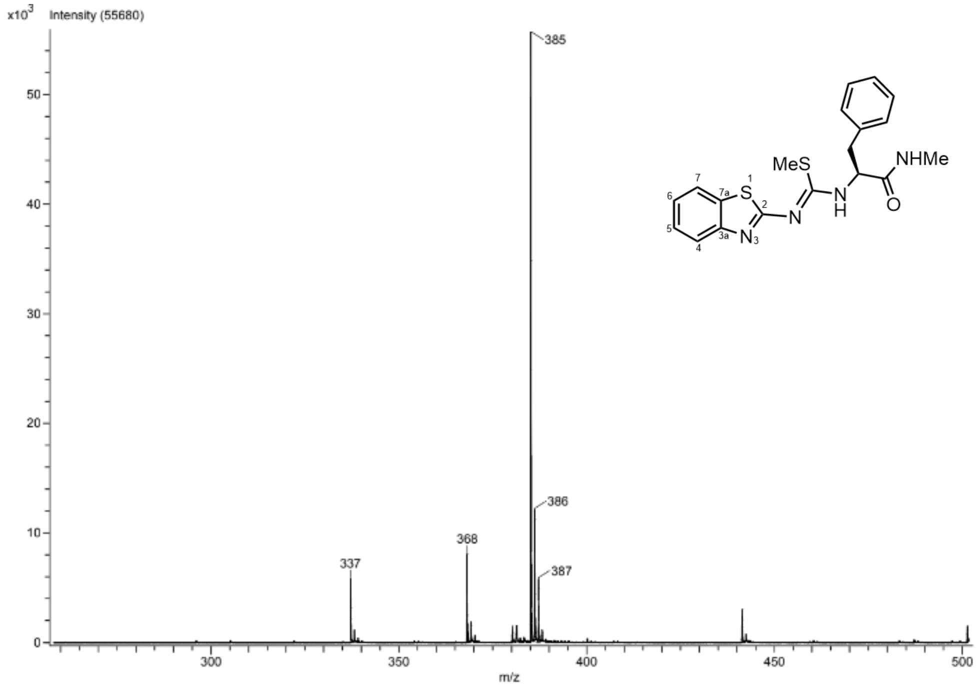

Figure S17. SM (DART+) of compound **12d.**

Description:
Ionization Mode:ESI+
History:Determine m/z[Peak Detect[Centroid,30,Area];Correct Base[5.0%]];Correct Base[5.0%];Average(MS[1] 1..1)

Mass Calibration data:CAL_PEG_600_ok
Created:5/21/2024 8:48:56 AM
Created by:AccuTOF

Charge number:1
Tolerance:3.00(mmu)
Unsaturation Number:-1.5 .. 15.0 (Fraction:.5)
Element:^{12}C:0 .. 30, ^{1}H:0 .. 60, ^{14}N:0 .. 5, ^{16}O:1 .. 3, ^{32}S:1 .. 2

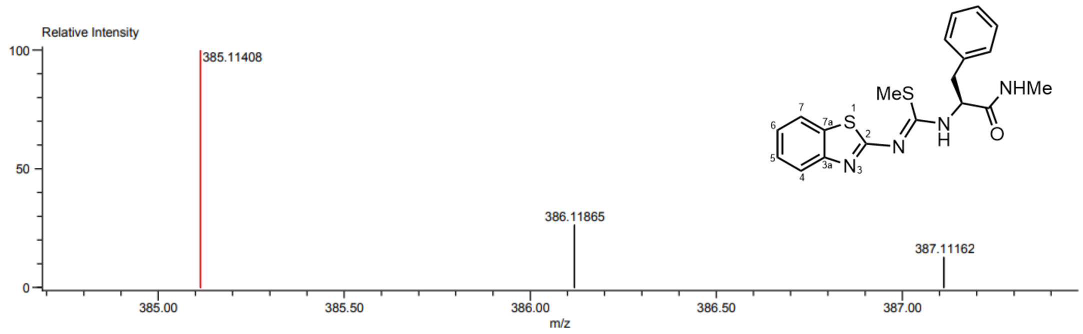

Mass	Intensity	Calc. Mass	Mass Difference (mmu)	Mass Difference (ppm)	Possible Formula	Unsaturation Number
385.11408	36380.95	385.11568	-1.60	-4.15	$^{12}C_{19}{}^{1}H_{21}{}^{14}N_{4}{}^{16}O_{1}{}^{32}S_{2}$	13.5

Figure S18. SM (ESI+) of compound **12d.**

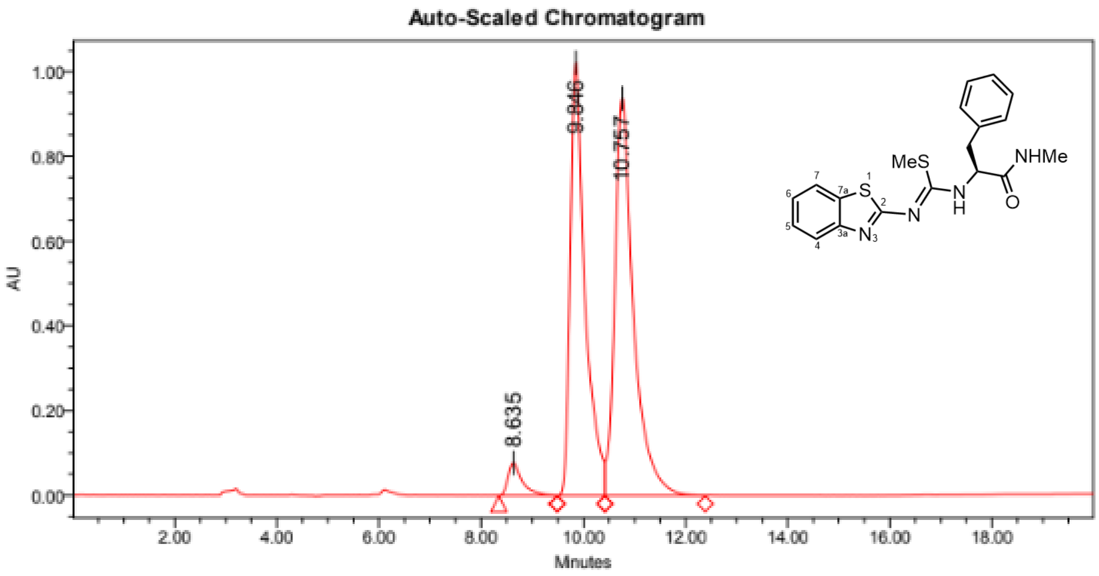

Peak Results

	Name	RT	Area	Height	Amount	Units
1		8.635	1473476	76636		
2		9.846	20558468	1022635		
3		10.757	23874895	938667		

PDA Result Table

	Name	RT	Purity1 Angle	Purity1 Threshold	Match1 Spect. Name	Match1 Angle	Match1 Threshold
1		8.635	0.734	0.390			
2		9.846	0.111	0.276			
3		10.757	0.168	0.275			

Figure S19. HPLC analysis of compound **12d.**

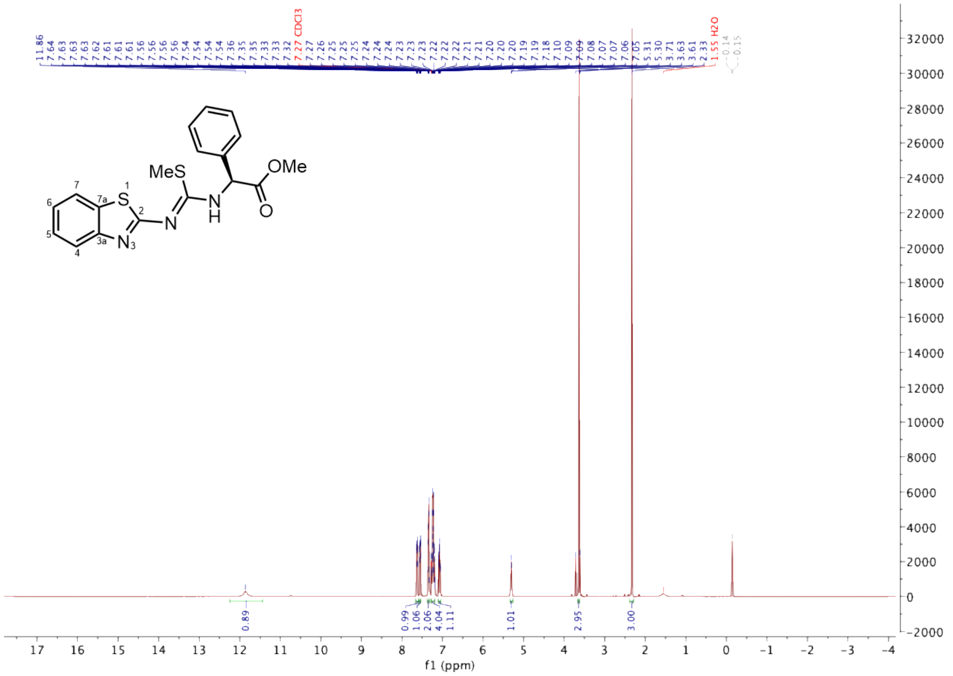

Figure S20. ^{1}H NRM (400 MHz, $CDCl_3$) of compound **8c**.

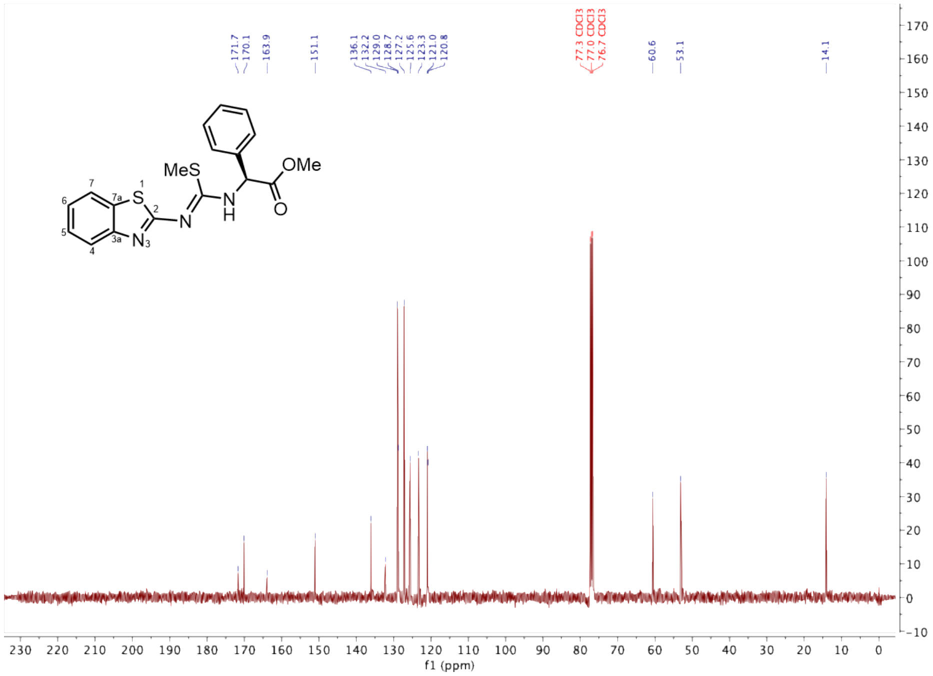

Figure S21. 13C NRM (101 MHz, CDCl3) of compound 8c.

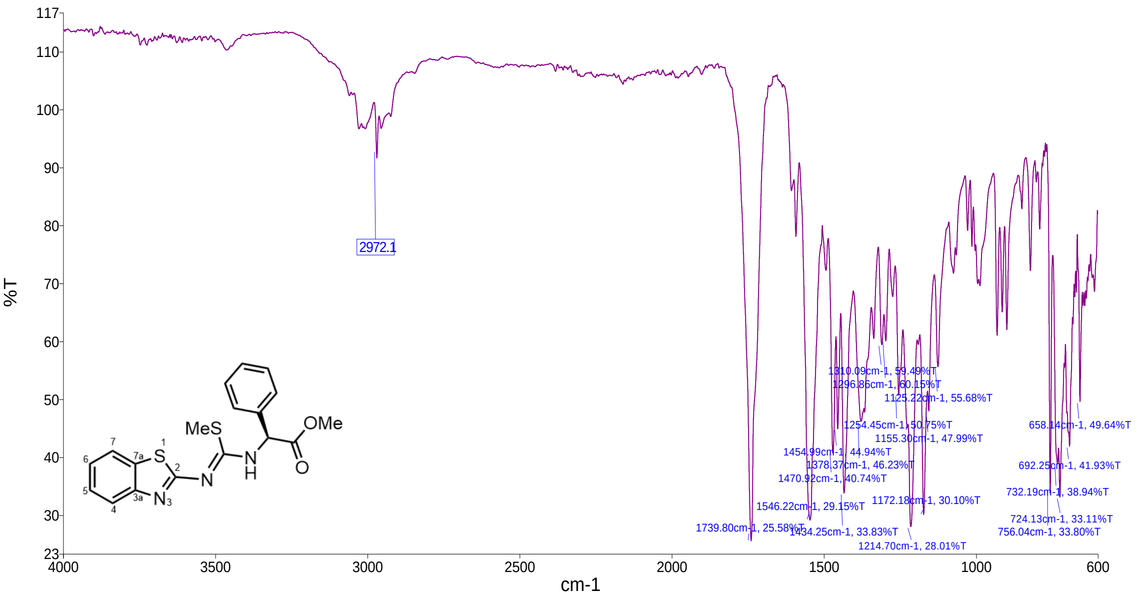

Figure S22. FTIR-ATR of compound **8c**.

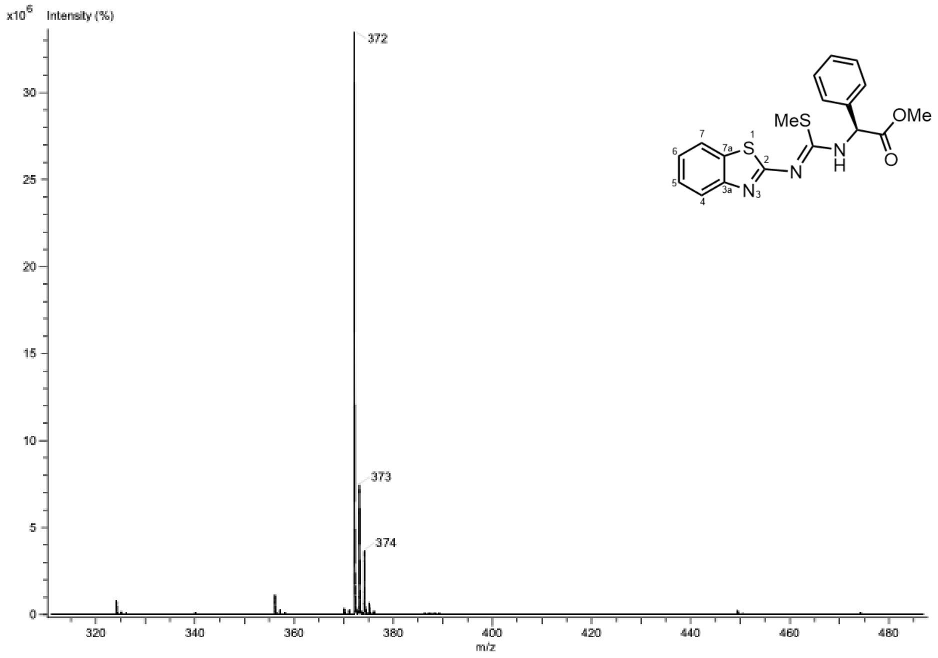

Figure S23. SM (DART+) of compound **8c**.

Description:
Ionization Mode:ESI+
History:Determine m/z[Peak Detect[Centroid,30,Area];Correct Base[5.0%]];Correct Base[5.0%];Average(MS[1] 1..1)

Mass Calibration data:CAL_PEG_600_JEOL_23-OCT-...
Created:11/12/2024 1:23:41 PM
Created by:AccuTOF

Charge number:1
Tolerance:1.00(ppm), 5.00 .. 15.00(mmu)
Unsaturation Number:0.0 .. 100.0 (Fraction:Both)
Element:^{12}C:0 .. 18, ^{1}H:0 .. 40, ^{14}N:0 .. 3, ^{16}O:0 .. 2, ^{32}S:0 .. 2

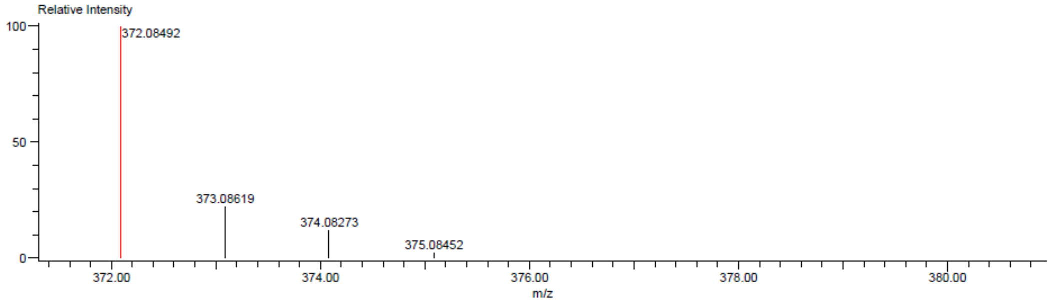

Mass	Intensity	Calc. Mass	Mass Difference (mmu)	Mass Difference (ppm)	Possible Formula	Unsaturation Number
372.08492	1923641.44	372.08404	0.88	2.37	$^{12}C_{18}{}^{1}H_{18}{}^{14}N_{3}{}^{16}O_{2}{}^{32}S_{2}$	11.5

Figure S24. SM (ESI+) of compound **8c**.

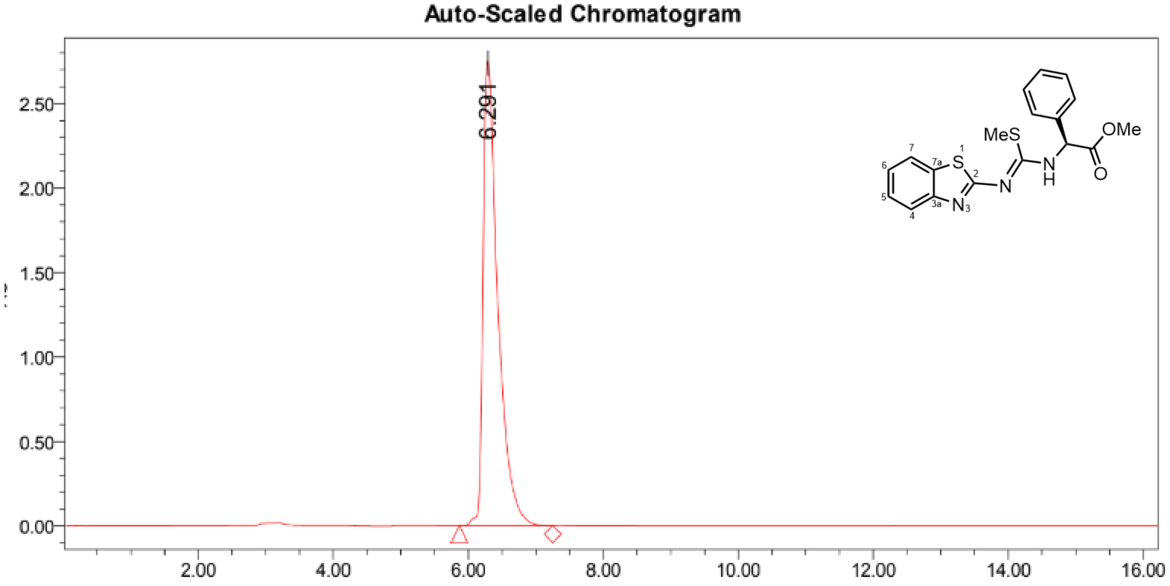

Figure S25. HPLC analysis of compound **8c**.

4. In vitro results

Table S6. Relative inhibition of the compounds tested on the ALR2 enzyme, measured from the rate of reduction of the NADPH cofactor with the kit (ab273276).

Inhibitor concentration	Epalrestat	8c	8d	12d
**10000 nm**	100.000	w/r	89.966	86.455
**5000 nm**	58.372	53.000	80.389	48.448
**500 nm**	12.513	68.371	86.494	33.993
**50 nm**	5.288	85.158	91.644	32.532
**5 nm**	5.000	82.945	93.590	29.534
**0.5 nm**	3.000	90.000	98.000	32.814

Data represented as percentage (%) relative ALR2 inhibition. c/r: no reading.

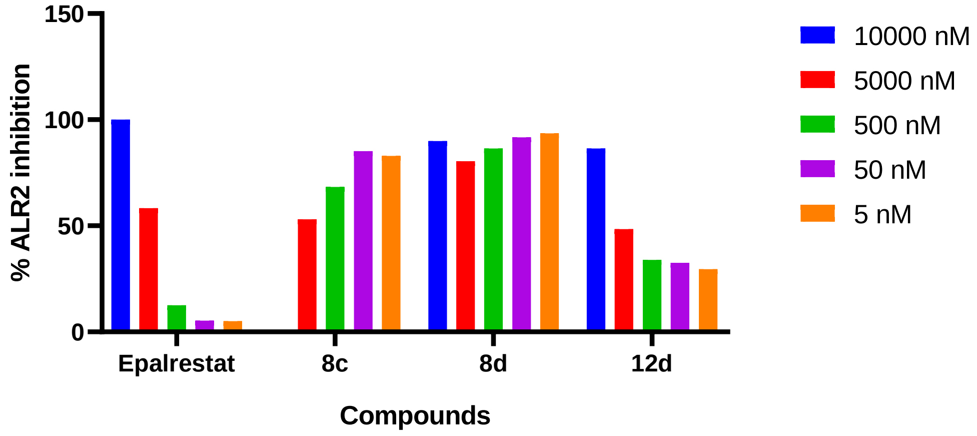

Figure S26. Comparison of the inhibition percentages. Data represented as percentage (%) relative ALR2 inhibition. c/r: no reading.

Table S7. Percentage reduction of ferric ion to ferrous ion in the presence of the different compounds. (FRAP assay)

Concentration	8c	8d	12d
**110 µM**	20.2	18.4	0.18
**90 µM**	19.6	16.9	0.20
**70 µM**	15.5	15.1	0.18
**50 µM**	14.4	14.1	0.20
**30 µM**	13.2	12.7	0.19

Data represented as percentage (%) of ferric ion reduction. Evaluation performed in triplicate.

Table S8. Percentage reduction of radical cation ABTS in the presence of the different compounds. (ABTS assay)

Concentration	8c	8d	12d
**110 µM**	-2.8	-2.5	-1.9
**90 µM**	-2.9	-3.6	-3.1
**70 µM**	-3.8	-4.2	-2.3
**50 µM**	-3.9	-3.0	-3.7
**30 µM**	-3.5	-4.0	-3.4

Data represented as percentage (%) reduction of the ABTS radical cation. Evaluation performed in triplicate.

5. In vivo results

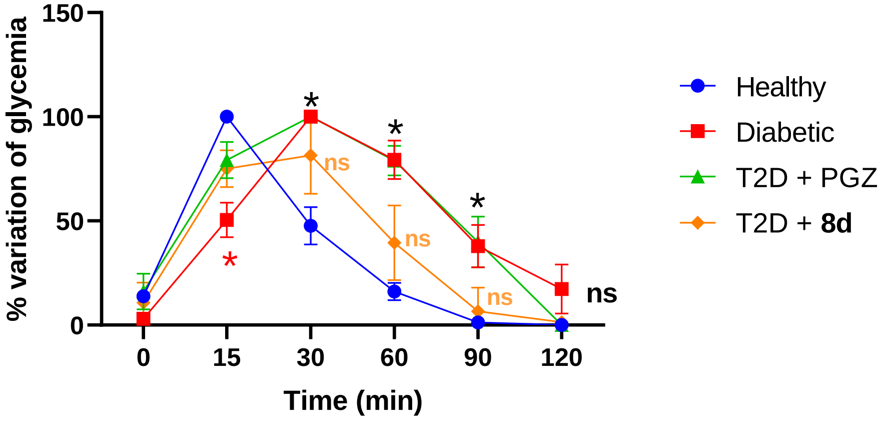

Figure S27. Oral glucose tolerance test (OGTT). Effect of treatments on blood glucose levels after a single oral administration of 1.5 g/Kg of glucose. Data reported as the mean ± SEM with $n = 4$. *Significant difference against healthy control ($p < 0.05$); ns: No significant difference against healthy control ($p > 0.05$).

Table S9. Values of the parameters measured in the 24-element blood chemistry.

	**Healthy (*n*=4)**		**Diabetic (*n*=4)**		**T2D + PGZ (*n*=4)**		**T2D + 8d (*n*=4)**	
	**Initial**	**Final**	**Initial**	**Final**	**Initial**	**Final**	**Initial**	**Final**
**TP**	6.45 ± 0.24	6.75 ± 0.09	5.93 ± 0.13	6.73 ± 0.19	6.5 ± 0.15	6.43 ± 0.22	6.73 ± 0.20	7.13 ± 0.28
**ALB**	3.925 ± 0.11	3.98 ± 0.03	3.60 ± 0.11	3.50 ± 0.16	3.85 ± 0.06	3.78 ± 0.12	3.78 ± 0.08	4.00 ± 0.16
**GLO**	2.525 ± 0.14	2.78 ± 0.07	2.33 ± 0.09	3.23 ± 0.19	2.65 ± 0.10	2.65 ± 0.12	2.95 ± 0.22	3.13 ± 0.13
**A/G**	1.55 ± 0.05	1.43 ± 0.03	1.58 ± 0.09	1.08 ± 0.09	1.45 ± 0.03	1.43 ± 0.05	1.3 ± 0.11	1.28 ± 0.03
**TBIL**	0.1975 ± 0.03	0.20 ± 0.03	0.185 ± 0.04	0.30 ± 0.11	0.2 ± 0.02	0.16 ± 0.02	0.31 ± 0.05	0.25 ± 0.05
**ALT**	77.5 ± 18.88	65.50 ± 10.04	61.25 ± 5.07	96.25 ± 28.10	63.5 ± 6.5	75.75 ± 6.73	68.75 ± 11.67	85.50 ± 27.46
**AST**	132.25 ± 6.50	327.00 ± 56.92#	114.75 ± 9.47	334.00 ± 56.12#	119.25 ± 7.96	343.50 ± 39.59#	135.75 ± 8.14	319.75 ± 51.70
**AST/ALT**	1.91 ± 0.3	4.96 ± 0.23	1.88 ± 0.12	4.04 ± 0.65	1.99 ± 0.38	4.61 ± 0.51	2.16 ± 0.40	4.42 ± 0.79
**GGT**	0.65 ± 0.42	0.38 ± 0.10	1.03 ± 0.3	0.80 ± 0.25	0.80 ± 0.25	1.25 ± 0.19	1.33 ± 0.38	0.65 ± 0.18
**ALP**	164.75 ± 16.01	116.25 ± 21.58	385.25 ± 75.79*	422.00 ± 114.73*	271.25 ± 44.69x	274.00 ± 58.19	287.75 ± 31.54*	210.25 ± 109.57
**TBA**	51.125 ± 6.54	33.8 ± 6.92	83.4 ± 18,92	102.90 ± 30.62	76.43 ± 13.25	72.60 ± 16.24	79.85 ± 13.64	54.33 ± 31.99
**CK**	625.75 ± 65.30	>3000 #	521 ± 90.85	>3000 #	509 ± 118.04 *	>3000 #	641.75 ± 63.37x	>3000 #
**AMY**	383.5 ± 33.37	355 ± 22.69	336.25 ± 24.24	317.00 ± 17.71	369.75 ± 26.22	405.75 ± 25.93	361.25 ± 6.54	436.50 ± 29.54
**TG**	104.75 ± 9.29	123.43 ± 2.82	229.6 ± 91.65 *	338.45 ± 136.01*	108.13 ± 6.46 x	151.00 ± 10.42	291.35 ± 160.5*	177.90 ± 82.61
**T-CHOL**	84.00 ± 2.04	92.00 ± 1.29	95.75 ± 15.63	118.50 ± 30.39	81.00 ± 1.96	98.00 ± 5.57	105.5 ± 19.94	90.00 ± 16.06
**GLU**	145.25 ± 5.62	90.00 ± 4.18	318.75 ± 35.8 *	316.00 ± 76.79 *	203.25 ± 19.84x	275.25 ± 84.75	188.50 ± 31.74x	172.75 ± 50.56
**CRE**	0.54 ± 0.01	0.58 ± 0.04	0.48 ± 0.01	0.56 ± 0.03	0.56 ± 0.03	0.54 ± 0.04	0.56 ± 0.00	0.62 ± 0.03
**UREA**	36.03 ± 2.46	38.83 ± 2.72	27.65 ± 4.11	52.08 ± 8.21	30.40 ± 3.28	31.58 ± 2.78	25.65 ± 3.10	38.10 ± 9.11
**BUN**	17.88 ± 0.06	18.15 ± 1.23	12.96 ± 1.89	24.28 ± 3.84	14.22 ± 1.51	14.81 ± 1.23	11.94 ± 1.46	17.78 ± 4.29
**BUN/CRE**	33.25 ± 0.75	31.50 ± 1.44	27.00 ± 3.87	42.75 ± 4.96	25.75 ± 2.59	28.00 ± 3.24	21.50 ± 2.60	28.50 ± 6.00
**tCO2**	14.25 ± 0.48	17.00 ± 0.41	15.00 ± 0.82	16.50 ± 0.65	13.75 ± 0.85	18.00 ± 0.41	15.25 ± 0.63	18.00 ± 0.82
**P**	8.44 ± 0.26	8.83 ± 0.26	7.08 ± 0.39	9.06 ± 0.41	7.55 ± 0.87	8.30 ± 0.47	6.83 ± 0.64	7.89 ± 0.55
**Ca**	9.50 ± 0.33	9.45 ± 0.13	9.38 ± 0.10	9.63 ± 0.27	9.30 ± 0.20	9.18 ± 0.08	9.45 ± 0.16	9.82 ± 0.48
**Mg**	2.36 ± 0.10	2.62 ± 0.06	1.94 ± 0.03	2.31 ± 0.14	2.09 ± 0.03	2.40 ± 0.05	2.18 ± 0.07	2.34 ± 0.09

Data represented as the mean ± SEM with $n = 4$. * Statistical significance versus baseline values of the healthy control ($p < 0.05$ versus Healthy Control); X Statistical significance versus baseline values of the diabetic control ($p < 0.05$ versus Diabetic Control); # Statistical significance of final values versus baseline values ($p < 0.05$ versus baseline values of the same group).

6. Ex vivo results

Table S10. Parameters obtained after the induction of T2D, before treatment.

	**Healthy**	**Diabetic**	**T2D + PGZ**	**T2D + 8d**
HbA1c	6.267 ± 0.291	10.133 ± 0.47	8.433 ± 0.088	8.633 ± 0.536
HOMA-IR	1.97 ± 0.438	2.68 ± 0.568	2.59 ± 0.337	4.22 ± 1.577
R-QUICKI	0.35 ± 0.015	0.334 ± 0.012	0.333 ± 0.007	0.318 ± 0.013

Data represented as the mean ± SEM with *n* = 4.

Table S11. Parameters obtained at the end of the evaluation period. After 5 weeks of treatment.

	**Healthy**	**Diabetic**	**T2D + PGZ**	**T2D + 8d**
HbA1c	6.4 ± 0.416	11.667 ± 0.561	9.633 ± 1.601	9.267 ± 0.802
HOMA-IR	1.41 ± 0.399	5.42 ± 1.849	2.32 ± 0.407	1.66 ± 0.611
R-QUICKI	0.378 ± 0.025	0.307 ± 0.012	0.339 ± 0.008	0.376 ± 0.031
TNF-α [pg/mL]	1641.41 ± 266.06	4452.61 ± 294.68	3000.53 ± 519.24	5417.72 ± 2104.18
IL-6 [pg/mL]	373.75 ± 130.19	536.82 ± 136.82	234.25 ± 52.05	887.53 ± 243.91
TAC [µM*]	113.29 ± 8.39	87.49 ± 22.28	97.75 ± 1.29	79.26 ± 17.28

Data represented as the mean ± SEM with *n* = 4; * equivalent to Trolox
